# Structure-activity relationships and molecular docking studies of chromene and chromene based azo chromophores: A novel series of potent antimicrobial and anticancer agents

**DOI:** 10.17179/excli2017-356

**Published:** 2017-06-19

**Authors:** Tarek H. Afifi, Rawda M. Okasha, Hany E.A. Ahmed, Janez Ilaš, Tarek Saleh, Alaa S. Abd-El-Aziz

**Affiliations:** 1 Chemistry Department, Faculty of Science, Taibah University, 30002, Al-Madinah Al-Munawarah, Saudi Arabia; 2 Pharmacognosy and Pharmaceutical Chemistry Department, Pharmacy College, Taibah University, Al-Madinah Al-Munawarah, Saudi Arabia; 3 Pharmaceutical Organic Chemistry Department, Faculty of Pharmacy, Al-Azhar University, Cairo, Egypt; 4 Faculty of Pharmacy, University of Ljubljana, Aškerceva 7, 1000 Ljubljana, Slovenia; 5 Department of Biomedical Sciences, Ontario Veterinary College, University of Guelph, Guelph, ON, N1G 2W1, Canada; 6 Chemistry Department, Faculty of Science, University of Prince Edward Island, Charlottetown, Prince Edward Island C1A 4P3, Canada

**Keywords:** chromene compounds, chromene azo dyes, antitumor activity, biological applications, 2D QSAR and docking simulations

## Abstract

The design of novel materials with significant biological properties is a main target in drug design research. Chromene compounds represent an interesting medicinal scaffold in drug replacement systems. This report illustrates a successful synthesis and characterization of two novel series of *chromene* compounds using multi-component reactions. The synthesis of the first example of azo chromophores containing chromene moieties has also been established using the same methodology. The antimicrobial activity of the new molecules has been tested against seven human pathogens including two Gm+ve, two Gm-ve bacteria, and four fungi, and the results of the inhibition zones with minimum inhibitory concentrations were reported as compared to reference drugs. All the designed compounds showed significant potent antimicrobial activities, among of them, four potent compounds **4b**, **4c**, **13e**, and **13i** showed promising MIC from 0.007 to 3.9 µg/mL. In addition, antiproliferative analysis against three target cell lines was examined for the novel compounds. Compounds **4a**, **4b**, **4c**, and **7c** possessed significant antiproliferative activity against three cell lines with an IC_50_ of 0.3 to 2 µg/mL. Apoptotic analysis was performed for the most potent compounds via caspase enzyme activity assays as a potential mechanism for their antiproliferative effects. Finally, the computational 2D QSAR and docking simulations were accomplished for structure-activity relationship analyses.

## Introduction

The vast majority of health problems facing the world's population at the present time are cancer and infectious diseases (Trinchieri, 2015[61]). Cancer is one of the most frequent causes of death in developing countries, and the identification of new therapies is an area of ongoing importance in biomedical research (Higginson and Costantini, 2008[24]; Varmus, 2006[63]). Liver, colon, breast, and melanoma cancers are most common in developed and underdeveloped countries. Therefore, there is an urgent need to search for new specific anticancer agents (Settimo et al., 1998[54]; Sondhi et al., 2010[58]). For this purpose, chemotherapy is the most commonly used treatment worldwide to cure various types of cancers (Isakoff, 2010[27]). Currently, combination chemotherapy with different mechanisms of action is one of the methods that are being adopted to treat cancer (Pritchard et al., 2013[47]).

Meanwhile, bacterial infections remain a serious threat to human lives because of its emerging resistance to existing antibiotics, which is another increasing public health problem. Consequently, there is a vital need for the development of new antimicrobial agents with potent activity against drug resistant microorganisms (Coates et al., 2002[15]). The drug discovery research of antimicrobial agents, accompanied by clinical development, has largely been conducted by many pharmaceutical companies (Bush, 2004[10]) in order to develop new safer, potent, and resistance-free antimicrobial drugs (Devasahayam et al., 2010[17]). For this reason, the discovery of novel anticancer and antimicrobial agents is important and timely endeavor for public health.

Oxygen atom-containing heterocyclic molecules are one of the main targets in drug design research. In particular, chromene compounds are well known as important components either in biologically active synthetic or natural compounds (Devakaram et al., 2012[16]; Iriti and Faoro, 2010[26]; Juan et al., 2001[29]; Ren et al., 2011[48]). Certain natural and synthetic chromene analogues have shown a diversity of interesting properties over the years (Ali et al., 2015[5]; Cai et al., 2006[11]; Cheng et al., 2003[14]; Jain et al., 2009[28]; Kamdar et al., 2010[30]; Kemnitzer et al., 2008[31]; Mladenovic et al., 2011[40]; Mori et al., 2006[41]; Thareja et al., 2010[60]). Some of these molecules exhibited significant effects as antitumor (Cai et al., 2006[11]; Kemnitzer et al., 2008[31]), antivascular (Gourdeau et al., 2004[23]), antimicrobial (Ali et al., 2015[5]), antioxidant (Mladenovic et al., 2011[40]), antifungal (Thareja et al., 2010[60]), anticoagulant (Jain et al., 2009[28]), anti-HIV (Park et al., 2008[45]) and anti-inflammatory (Kamdar et al., 2010[30]) activities. The most important character of these molecules is the lipophilic character of the benzopyran moieties which facilitates their delivery into the cell membrane (Nicolaou et al., 2000[44]).

Chromene compounds have been synthesized using various approaches, including multi-component reactions (MCRs) (Boominathan et al., 2011[9]; Elinson et al., 2010[20]; Kirilmis et al., 2008[35]; Shafiei-Haghighi, 2011[55]), heterogeneous catalytic methods (Mehrabi and Kazemi-Mireki, 2011[39]; Yadav et al., 2007[67]), electro-catalytic process (Makarem et al., 2008[38]), green synthesis routes using aqueous media (Kumar et al., 2009[37]; Murthy et al., 2010[43]), microwave (Sangani et al., 2012[53], 2013[52]) and ultrasound techniques (Safari and Javadian, 2015[51]). In addition, chromene compounds seem to be interesting precursors for the preparation of azo dyes, either as amine or coupler molecules. Azo chromophores synthesis and developments is a very rich area in current literature (Abd-El-Aziz and Afifi, 2006[1]; Shawali and Samy, 2015[56]), however, this topic still inspires many researchers due to the new possibilities of applications that this class of compounds may possess (Abd-El-Aziz and Afifi, 2006[1]; Shawali and Samy, 2015[56]; Wainwright, 2008[65]; Zheng et al., 2010[68]). Due to the high biological activity of the chromene compounds as well as the interesting optical properties of azo molecules, combination of these molecules allows the construction of new materials that expected to reveal new chemical, physical as well as biological properties.

Meanwhile, it is well known that the most important aspect of activity of 4-Aryl-4H-chromenes is the apoptotic modulation effect by cell- and caspase-based protocols on cancer cells, leading to growth inhibition and hence cell death (Kemnitzer et al., 2004[32], 2005[34], 2007[33]). For instance, Kemnitzer et al. (2008[31]) have reported 4-aryl-4H-chromenes as potent inducers of apoptosis through tubulin inhibition. They indicated that the 4-position is quite critical for the structure-activity relationship of such derivatives.

In view of the above mentioned benefits and in continuation of our previous work in development synthesis of polyfunctional-substituted heterocyclic compounds with potential biological activity (Abd-El-Aziz et al., 2016[2]; Ahmed et al., 2016[4]), we report here the utility of the 3- and 5-aminonaphthol components as building blocks with different physicochemical properties for the synthesis of 2,7-diamino-4-phenyl-4H-benzo[h]chromene-3-carbonitrile (**4a-i**) and 3,5-diamino-1-phenyl-1H-benzo[f]chromene-2-carbonitrile (**7a-e**). In addition, the present study shows the synthesis of 2-amino-7-hydroxy-4-phenyl-6-(phenyldiazenyl)-4H-chromene-3-carbonitrile (**13a-k**) compounds as a first example of azo dye incorporating chromene derivatives with the purpose of investigating their possible biological and cytotoxic activities. Structures of the novel molecules were elucidated on the basis of IR, ^1^H-, ^13^C- NMR and MS, and their UV-vis behaviors were also examined.

### Study rationale

**Figure 1:**
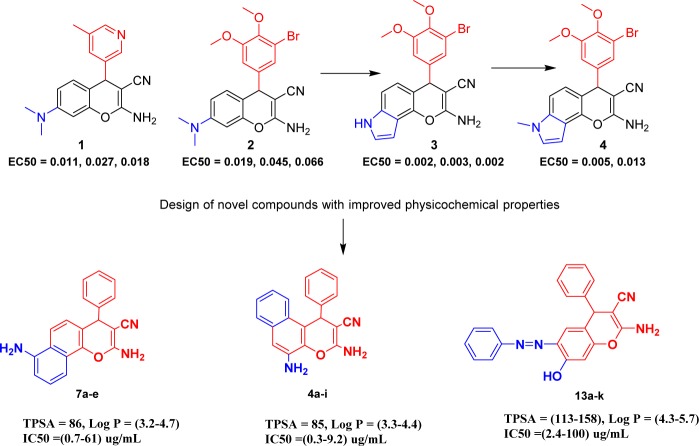
Target scaffold with corresponding novel series compounds appearing the mapping of physicochemical properties to biological activity

In continuation to our previous work, lipophilic parameters were found to control the activity of phenyl chromene carbonitrile derivatives (Du et al., 2011[18]; Gellert et al., 1976[22]; Widelski et al., 2009[66]). Accordingly, in our synthesis we consider the incorporation of fused aromatic systems in this scaffold to study the effect of the activity by using naphthyl amine precursors. In addition, we also demonstrated the formation of chromene azo dye via the formation of diazonium salt and its coupling with resorcinol to produce the stable azo dye. This reaction is followed by building the chromene scaffold with more polar surface area and lipophilic characters in order to increase the surface area in an effort to reach the maximum logP value as an indicator of lipophilicity (Figure 1(Fig. 1)).

## Experimental Work

### Chemistry

#### Materials & Characterization

All chemicals were available from Sigma-Aldrich Chemical Co. and were used without purification. Solvents were HPLC grade and were used without further purification, with the exception of ethanol, which was distilled prior to use. 2,7-amino-4-aryl-3-cyano-4H-chromene and 2-amino-4-aryl-7-hydroxy-3-cyano-4H-chromene series were synthesized according to a previous report (Voskressensky et al., 2014[64]). The progress of the reactions was monitored using thin layer chromatography (TLC) on Merck silica gel 60 F254 plates. ^1^H and ^13^C NMR spectra were recorded at 400 and 125 MHz, respectively, on a Gemini 200 NMR spectrometer, with chemical shifts calculated in Hz, referenced to solvent residues. Infrared spectra were recorded on a Bomem, Hartmann & Braun FT-IR spectrophotometer as KBr pellets. Mass spectra were measured using a Shimadzu GC/MS-QP5050A spectrometer (Shimadzu, Japan). UV-vis measurements were performed on Carry 100. 

#### Preparation of 3,5-diamino-4-aryl-4H-benzochromene-2-carbonitriles (4a-i)

A mixture of 3-amino-2-naphthol (10 mmol), malononitrile (10 mmol) and aromatic aldehyde (10 mmol) were dissolved in EtOH (15 ml), then piperidine (0.5 ml) was added. The mixture was refluxed for 2-4 hours until complete precipitation occurred. The isolated precipitate was filtered, washed with ethanol and recrystallized from ethanol. Characterization of the new compounds was achieved using NMR, FTIR and mass spectrometry.

**3,5-diamino-1-(4-fluorophenyl)-1*H*-benzo[*f*]chromene-2-carbonitrile (4a)**

Colorless crystals from ethanol (67 % yield), mp 193 °C; IR (KBr) cm^-1^: 3320, 3425, 3490 (NH_2_), 2190 (CN), 3140 (CH); ^1^H NMR (400 MHz) (DMSO-d_6_) δ (ppm): 5.20 (s, 1H, H-4), 5.45 (s, 2H, NH_2_-10) 6.85 (s, 2H, NH_2_-2), 6.91 (s, 1H, Ar-H), 6.97-7.04 (m, 3H, Ar-H), 7.13-7.17 (m, 3H, Ar-H), 7.47 (d, *J*= 9.0 Hz, 1H, Ar-H), 7.53 (d, *J*= 9.0 Hz, 1H, Ar-H). ^13^C NMR (125 MHz) (DMSO-d_6_) δ (ppm): 37.41(C-4), 57.98 (C-3), 106.85 (C-9), 115.52 (Ar-CH), 115.88 (C-4a), 120.37 (CN), 122.08 (C-5), 122.64 (C-4b), 123.21 (C-6) 124.89 (C-7), 125.66 (C-8), 128.69 (Ar-CH), 132.07 (C-8a), 136.29 (C-10), 138.05 (Ar-C), 141.83 (C-10a), 159.79 (C-2), 161.99 (Ar-C). ^19^F NMR (DMSO-d_6_) δ (ppm): -116.11 (Ar-F). MS m\z (%): 331 (M^+^, 20.5) with base peak at 237.

**3,5-diamino-1-(4-chlorophenyl)-1*H*-benzo[*f*]chromene-2-carbonitrile(4b)**

Colorless crystals from ethanol (73 % yield), mp 187 °C; IR (KBr) cm^-1^: 3320, 3425, 3490 (NH_2_), 2195 (CN), 3140 (CH); ^1^H NMR (400 MHz) (DMSO-d_6_) δ (ppm): 5.35 (s, 1H, H-4), 5.66 (s, 2H, NH_2_-10) 7.06-7.13 (m, 4H, NH_2_-2 & Ar-H), 7.23-7.29 (m, 3H, Ar-H), 7.35 (d, *J*= 9.0 Hz, 2H, Ar-H), 7.60 (d, *J*= 9.0 Hz, 1H, Ar-H), 7.64 (d, *J*= 9.0 Hz, 1H, Ar-H). ^13^C NMR (125 MHz) (DMSO-d_6_) δ (ppm): 37.46 (C-4), 57.78 (C-3), 107.11 (C-9), 115.57 (C-4a), 120.43 (CN), 122.22 (C-5), 122.72 (C-6), 123.19 (C-7) 124.98 (C-8), 125.74 (C-4b), 128.71 (Ar-CH), 131.23 (C-8a), 132.13 (Ar-C), 136.30 (C-10), 138.19 (Ar-C), 144.54 (C-10a), 159.89 (C-2). MS m\z (%): 347 (M^+^, 19.8) with base peak at 236 (100).

**3,5-diamino-1-(p-tolyl)-1*H*-benzo[*f*]chromene-2-carbonitrile(4c)**

Colorless crystals from ethanol (64 % yield), mp 180 °C; IR (KBr) cm^-1^: 3320, 3425, 3490 (NH_2_), 2198 (CN), 3140 (CH); ^1^H NMR (400 MHz) (DMSO-d_6_) δ (ppm): 2.12 (s, 3H, CH_3_), 5.09 (s, 1H, H-4), 5.43 (s, 2H, NH_2_-10) 6.79 (s, 2H, Ar-H), 6.90 (s, 1H, Ar-H), 6.98 (br s, 5H, NH_2_-2 & Ar-H), 7.13 (t, *J *= 9.0 Hz, 1H, Ar-H), 7.45 (d, *J *= 9.0 Hz, 1H, Ar-H), 7.52 (d, *J *= 9.0 Hz, 1H, Ar-H). ^13^C NMR (125 MHz) (DMSO-d_6_) δ (ppm): 20.58 (CH_3_), 37.98 (C-4), 58.28 (C-3), 106.74 (C-9), 116.17 (C-4a), 120.54 (CN), 122.02 (C-5), 122.82 (C-6), 123.33 (C-7), 124.83 (Ar-C), 125.64 (C-8), 126.81 (Ar-CH), 129.24 (Ar-CH), 132.08 (C-4b), 135.66 (C-8a), 136.28 (C-10), 138.09 (Ar-C), 142.71 (C-10a), 159.71 (C-2). MS m\z (%): 327 (M^+^, 15.9) with base peak at 236 (100)

**3,5-diamino-1-(4-methoxy-phenyl)-1*H*-benzo[*f*]chromene-2-carbonitrile(4d)**

Colorless crystals from ethanol (60 % yield), mp 183 °C; IR (KBr) cm^-1^: 3320, 3425, 3490 (NH_2_), 2185 (CN), 3140 (CH); ^1^H NMR (400 MHz) (DMSO-d_6_) δ (ppm): 3.67 (s, 3H, CH_3_), 5.17 (s, 1H, H-4), 5.51 (s, 2H, NH_2_-10), 6.82 (d, *J *= 9.0 Hz, 2H, Ar-H), 6.87 (s, 2H, NH_2_-2), 6.98 (br. s, 1H, Ar-H), 7.05-7.12 (m, 3H, Ar-H), 7.22 (t, *J *= 9.0 Hz, 1H, Ar-H), 7.54 (d, *J*= 9.0 Hz, 1H, Ar-H), 7.63 (d, *J*= 9.0 Hz, 1H, Ar-H). ^13^C NMR (125 MHz) (DMSO-d_6_) δ (ppm): 37.50 (C-4), 55.60 (OCH_3_), 58.34 (C-3), 107.00 (C-9), 116.19 (C-4a), 120.40 (CN), 122.09 (C-5), 122.87 (C-6), 123.34 (C-7) 124.88 (C-8), 125.64 (C-4b), 126.89 (C-8a), 128.74 (Ar-CH), 138.17 (C-10), 146.46 (C-10a), 159.81 (Ar-C), 160.07 (C-2). MS m\z (%): 343 (M^+^, 13.95) with base peak at 186 (100). 

**3,5-Diamino-1-(2,4-dichloro-phenyl)-1*H*-benzo[*f*]chromene-2-carbonitrile (4e)**

Colorless crystals from ethanol (69 % yield), mp 278 °C; IR (KBr) cm^-1^: 3320, 3425, 3490 (NH_2_), 2200 (CN), 3140 (CH); ^1^H NMR (400 MHz) (DMSO-d_6_) δ (ppm): 5.57 (s, 2H, NH_2_-10), 5.67 (s, 1H, H-4), 6.99 (d, *J*= 9.0 Hz, 1H, Ar-H), 7.05 (br. s, 3H, Ar-H & NH_2_-2), 7.10 (d, *J*= 9.0 Hz, 1H, Ar-H), 7.19-7.23 (m, 2H, Ar-H), 7.38 (d, *J*= 9.0 Hz, 1H, Ar-H), 7.56 (d, *J*= 9.0 Hz, 1H, Ar-H), 7.59 (s, 1H, Ar-H). ^13^C NMR (125 MHz) (DMSO-d_6_) δ (ppm): 35.29 (C-4), 56.63 (C-3), 107.82 (C-9), 115.05 (C-4a), 120.17 (CN), 122.61 (C-5), 122.94 (C-6), 123.06 (C-4b), 125.48 (C-7) 126.34 (C-8), 128.90 (Ar-CH), 129.21 (Ar-CH), 131.57 (Ar-CH), 132.22 (C-8a), 132.47 (Ar-C), 136.64 (C-10), 138.88 (Ar-C), 142.16 (C-10a), 160.37 (C-2). ^19^F NMR (DMSO-d_6_) δ (ppm): -111.92, -115.30 (Ar-F). MS m\z (%): 382 (M^+^, 12.5) with base peak at 237.

**3,5-Diamino-1-(2,4-difluoro-phenyl)-1*H*-benzo[*f*]chromene-2-carbonitrile (4f)**

Colorless crystals from ethanol (69 % yield), mp 282 °C; IR (KBr) cm^-1^: 3320, 3425, 3490 (NH_2_), 2202 (CN), 3140 (CH); ^1^H NMR (400 MHz) (DMSO-d_6_) δ (ppm): 5.49 (s, 1H, H-4), 5.54 (s, 2H, NH_2_-10), 6.95 (t, *J*= 9.0 Hz, 1H, Ar-H), 7.03 (br. s, 3H, Ar-H & NH_2_-2), 7.10 (t, *J*= 9.0 Hz, 1H, Ar-H), 7.18-7.22 (m, 3H, Ar-H), 7.50-7.56 (m, 2H, Ar-H). ^13^C NMR (125 MHz) (DMSO-d_6_) δ (ppm): 32.69 (C-4), 56.57 (C-3), 104.50 (Ar-CH), 107.61 (C-9), 114.66 (C-4a), 120.59 (CN), 122.69 (C-6), 122.79 (C-7), 123.04 (C-8), 125.38 (C-5) 126.29 (Ar-CH), 128.74 (C-4b), 128.88 (C-8a), 131.21 (Ar-C), 132.46 (Ar-CH), 136.67 (C-10), 138.89 (C-10a), 158.55 (Ar-C), 160.32 (Ar-C), 160.64 (C-2). ^19^F NMR (DMSO-d_6_) δ (ppm): -111.92, -115.30 (Ar-F). MS m\z (%): 349 (M^+^, 18.9) with base peak at 237.

**3,5-Diamino-1-(2-fluoro-3-trifluoromethyl-phenyl)-1*H*-benzo[*f*]chromene-2-carbonitrile (4g)**

Colorless crystals from ethanol (64 % yield), mp 202 °C; IR (KBr) cm^-1^: 3320, 3425, 3490 (NH_2_), 2210 (CN), 3140 (CH); ^1^H NMR (400 MHz) (DMSO-d_6_) δ (ppm): 5.57 (s, 2H, NH_2_-10), 5.63 (s, 1H, H-4), 7.05 (s, 2H, NH_2_-2), 7.11 (br. s, 3H, Ar-H), 7.21-7.29 (m, 2H, Ar-H), 7.51-7.57 (m, 4H, Ar-H). ^13^C NMR (125 MHz) (DMSO-d_6_) δ (ppm): 33.31(C-4), 56.06 (C-3), 107.86 (C-9), 114.12 (C-F_3_), 120.44 (C-4a), 122.61 (CN), 122.98 (C-5), 124.45 (Ar-C), 125.47 (C-6) 125.76 (C-7), 126.37 (Ar-CH & C-8), 132.49 (Ar-CH), 133.97 (C-4b), 134.09 (C-8a), 135.05 (Ar-H), 136.70 (C-10), 138.98 (C-10a), 155.59 (Ar-C), 158.12 (Ar-C), 160.80 (C-2). ^19^F NMR (DMSO-d_6_) δ (ppm): -122.89 (Ar-F). MS m\z (%): 399 (M^+^, 12.6) with base peak at 237.

**3,5-Diamino-1-(3,4,5-trimethoxy-phenyl)-1*H*-benzo[*f*]chromene-2-carbonitrile (4h)**

Colorless crystals from ethanol (77 % yield), mp 225 °C; IR (KBr) cm^-1^: 3320, 3425, 3490 (NH_2_), 2198 (CN), 3140 (CH); ^1^H NMR (400 MHz) (DMSO-d_6_) δ (ppm): 3.61 (s, 3H, OCH_3_), 3.66 (s, 6H, OCH_3_), 5.24 (s, 1H, H-4), 5.52 (s, 2H, NH_2_-10), 6.53 (s, 2H, Ar-H), 6.94 (s, 2H, NH_2_-2), 7.03 (s, 1H, Ar-H), 7.10 (t, *J*= 9.0 Hz, 1H, Ar-H), 7.23 (t, *J*= 9.0 Hz, 1H, Ar-H), 7.56 (d, *J*= 9.0 Hz, 1H, Ar-H), 7.73 (d, *J*= 9.0 Hz, 1H, Ar-H). ^13^C NMR (125 MHz) (DMSO-d_6_) δ (ppm): 39.08 (C-4), 56.30 (OCH_3_), 58.66 (C-3), 60.41 (OCH_3_), 104.80 (Ar-CH), 107.52 (C-9), 116.38 (C-4a), 121.06 (CN), 122.65 (C-5), 123.52 (C-6) 123.88 (Ar-C), 125.42 (C-7), 126.14 (C-8), 132.54 (C-4b), 136.70 (C-8a), 138.70 (C-10), 141.85 (C-10a), 153.47 (Ar-C), 160.41 (C-2). MS m\z (%): 403 (M^+^, 21.5) with base peak at 237.

**3,5-Diamino-1-thiophen-3-yl-1*H*-benzo[*f*]chromene-2-carbonitrile (4i)**

Colorless crystals from ethanol (71 % yield), mp 230 °C; IR (KBr) cm^-1^: 3320, 3425, 3490 (NH_2_), 2196 (CN), 3140 (CH); ^1^H NMR (400 MHz) (DMSO-d_6_) δ (ppm): 5.38 (s, 1H, H-4), 5.50 (s, 2H, NH_2_-10), 6.84 (s, 1H, Ar-H), 6.95 (s, 2H, NH_2_-2), 7.01 (s, 1H, Ar-H), 7.12 (t, *J*= 9.0 Hz, 1H, Ar-H), 7.23-7.25 (m, 2H, Ar-H), 7.35 (br. s, 1H, Ar-H), 7.56 (d, *J*= 9.0 Hz, 1H, Ar-H), 7.78 (d, *J*= 9.0 Hz, 1H, Ar-H). ^13^C NMR (125 MHz) (DMSO-d_6_) δ (ppm): 33.94 (C-4), 57.92 (C-3), 107.26 (C-9), 116.89 (C-4a), 121.05 (CN), 121.09 (Ar-CH), 122.52 (C-5), 123.25 (C-6) 123.54 (C-4b), 125.32 (C-7), 126.05 (Ar-CH), 127.00 (C-8), 127.11 (Ar-CH), 132.43 (C-8a), 136.66 (C-10), 138.29 (Ar-C), 146.33 (C-10a), 160.89 (C-2). MS m\z (%): 319 (M^+^, 18.6) with base peak at 237.

#### Preparation of 2,7-diamino-4-aryl-4H-benzochromene-2-carbonitrile (7a-e)

A mixture of 5-amino-1-naphthol (10 mmol), malononitrile (10 mmol) and aromatic aldehyde (10 mmol) were dissolved in EtOH (15 ml), then piperidine (0.5 ml) was added. The mixture was refluxed for 2-4 hours until complete precipitation occurred. The isolated precipitate was filtered, washed with ethanol and recrystallized from ethanol. Characterization of the new compounds was achieved using NMR, FTIR and mass spectrometry.

**2,7-Diamino-4-(4-tert-butyl-phenyl)-4*H*-benzo[*h*]chromene-3-carbonitrile (7a)**

Gray crystals from ethanol (75 % yield), mp 210 °C; IR (KBr) cm^-1^: 3320, 3425, 3490 (NH_2_), 2190 (CN), 3140 (CH); ^1^H NMR (400 MHz) (DMSO-d_6_) δ (ppm): 1.23 (s, 9H, CH_3_), 4.80 (s, 1H, H-4), 5.76 (s, 2H, NH_2_-8) 6.71 (d, *J*= 9.0 Hz, 1H, Ar-H), 6.95 (d, *J*= 9.0 Hz, 1H, Ar-H), 7.05 (s, 2H, NH_2_-2), 7.15 (d, *J*= 9.0 Hz, 2H, Ar-H), 7.26-7.34 (m, 3H, Ar-H), 7.44 (d, *J*= 9.0 Hz, 1H, Ar-H), 7.75 (d, *J*= 9.0 Hz, 1H, Ar-H). ^13^C NMR (125 MHz) (DMSO-d_6_) δ (ppm): 31.09 (CH_3_), 34.10 (C-CH_3_), 40.48 (C-4), 56.18 (C-3), 107.99 (C-7), 108.34 (C-5), 117.64 (CN), 118.62 (C-4a), 120.77 (C-10), 122.02 (C-8a), 123.43 (C-9), 124.05 (C-6), 125.38, 127.10 (Ar-CH), 127.41 (C-4b), 142.91 (C-8), 144.87 (Ar-C), 149.04 (C-10a), 160.38 (C-2). MS m\z (%): 369 (M^+^, 18.6) with base peak at 237.

**2,7-Diamino-4-(4-methoxy-phenyl)-4*H*-benzo[*h*]chromene-3-carbonitrile (7b)**

Gray crystals from ethanol (75 % yield), mp 185 °C; IR (KBr) cm^-1^: 3320, 3425, 3490 (NH_2_), 2190 (CN), 3140 (CH); ^1^H NMR (400 MHz) (DMSO-d_6_) δ (ppm): 3.71 (s, 3H, OCH_3_), 4.79 (s, 1H, H-4), 5.78 (s, 2H, NH_2_-8), 6.72 (d, *J*= 9.0 Hz, 1H, Ar-H), 6.87 (d, *J*= 9.0 Hz, 2H, Ar-H), 6.92 (d, *J*= 9.0 Hz, 1H, Ar-H), 7.05 (s, 2H, NH_2_-2), 7.15 (d, *J*= 9.0 Hz, 2H, Ar-H), 7.30 (t, *J*= 9.0 Hz, 1H, Ar-H), 7.45 (d, *J*= 9.0 Hz, 1H, Ar-H), 7.74 (d, *J*= 9.0 Hz, 1H, Ar-H). ^13^C NMR (125 MHz) (DMSO-d_6_) δ (ppm): 40.52 (C-4), 55.44 (OCH_3_), 56.84 (C-3), 108.43 (C-7), 108.79 (C-5), 114.41 (Ar-CH), 118.15 (C-10), 118.99 (CN), 121.18 (C-4a), 122.41(C-9), 123.89 (C-8a), 124.47 (C-4b), 127.88 (C-6), 129.09 (Ar-CH), 138.45 (Ar-C), 143.18 (C-8), 145.33 (C-10a), 158.53 (Ar-C), 160.60 (C-2). MS m\z (%): 343 (M^+^, 18.6) with base peak at 237.

**2,7-Diamino-4-(2,4,6-trifluoro-phenyl)-4*H*-benzo[*h*]chromene-3-carbonitrile (7d)**

Greenish crystals from ethanol (52 % yield), mp 235 °C; IR (KBr) cm^-1^: 3320, 3425, 3490 (NH_2_), 2190 (CN), 3140 (CH); ^1^H NMR (400 MHz) (DMSO-d_6_) δ (ppm): 5.10 (s, 1H, H-4), 7.16 (d, *J*= 9.0 Hz, 1H, Ar-H), 7.29 (s, 2H, NH_2_-8), 7.38 (d, *J*= 9.0 Hz, 1H, Ar-H), 7.48 (d, *J*= 9.0 Hz, 2H, Ar-H), 7.67-7.72 (m, 3H, Ar-H & NH_2_-2), 7.93 (d, *J*= 9.0 Hz, 1H, Ar-H), 8.24 (d, *J*= 9.0 Hz, 1H, Ar-H). ^13^C NMR (125 MHz) (DMSO-d_6_) δ (ppm): 40.91 (C-4), 55.92 (C-3), 114.75 (C-7), 118.02 (Ar-CH), 119.44 (CN), 120.17 (C-6), 120.59 (C-5), 122.78 (C-4a), 123.76 (C-10), 126.05 (C-8a), 127.58 (C-9), 128.18 (C-6), 128.91, 129.88 (Ar-CH), 139.84 (C-4b), 143.33 (C-8), 148.12 (Ar-C), 150.49 (C-10a), 160.62 (C-2), 160.88 (Ar-C); ^19^F NMR (DMSO-d_6_) δ (ppm): -116.11 (Ar-F). MS m\z (%): 367 (M^+^, 12.6) with base peak at 237.

**2,7-Diamino-4-thiophen-2-yl-4*H*-benzo[*h*]chromene-3-carbonitrile (7e)**

Gray crystals from ethanol (64 % yield), mp 232 °C; IR (KBr) cm^-1^: 3320, 3425, 3490 (NH_2_), 2190 (CN), 3140 (CH), ^1^H NMR (400 MHz) (DMSO-d_6_) δ (ppm): 5.21 (s, 1H, H-4), 5.80 (s, 2H, NH_2_-8), 6.74 (d, *J*= 9.0 Hz, 1H, Ar-H), 6.95 (t, *J*= 9.0 Hz, 1H, Ar-H), 7.07-7.12 (m, 3H, Ar-H), 7.17 (s, 2H, NH_2_-2), 7.30 (t, *J*= 9.0 Hz, 1H, Ar-H), 7.37 (d, *J*= 9.0 Hz, 1H, Ar-H), 7.44 (d, *J*= 9.0 Hz, 1H, Ar-H), 7.81 (d, *J*= 9.0 Hz, 1H, Ar-H). ^13^C NMR (125 MHz) (DMSO-d_6_) δ (ppm): 37.02 (C-4), 57.17 (C-3), 107.99 (C-7), 108.34 (C-5), 117.64 (CN), 118.62 (C-4a), 120.77 (C-10), 122.02 (C-8a), 123.43 (C-9), 124.05 (C-6), 125.38, 127.10 (Ar-CH), 127.41 (C-4b), 142.91 (C-8), 144.87 (Ar-C), 149.04 (C-10a), 160.38 (C-2). MS m\z (%): 319 (M^+^, 18.6) with base peak at 237.

#### Preparation of chromene-based azo dyes

##### Synthesis of resorcinol azo dyes (12a-k) 

Azo dye compounds were prepared using previously reported method (Afifi, 2003[3]). 

**4-(p-tolyldiazenyl)-benzene-1,3-diol (12a)**

Orange crystals from ethanol (86 % yield), mp 185 °C; IR (KBr) cm^-1^: 3310 (OH), 1473 (N=N); ^1^H NMR (400 MHz) (DMSO-d_6_) δ (ppm): 2.42 (s, 3H, CH_3_), 6.38 (s, 1H, Ar-H), 6.54 (d, *J*= 9.0 Hz, 1H, Ar-H), 7.29 (d, *J*= 9.0 Hz, 2H, Ar-H), 7.65-7.69 (m, 3H, Ar-H), 10.04 (br s, 1H, OH), 13.61 (s, 1H, OH). ^13^C NMR (125 MHz) (DMSO-d_6_) δ (ppm): 21.30 (CH_3_), 103.29, 109.08, 121.28, 129.84, 132.18 (Ar-CH), 134.54, 140.18, 148.00, 156.05, 162.53 (Ar-C). 

**4-(4-Methoxy-phenylazo)-benzene-1,3-diol (12b)**

Orange crystals from ethanol (84 % yield), mp 187 °C; IR (KBr) cm^-1^: 3325 (OH), 1500 (N=N); ^1^H NMR (400 MHz) (DMSO-d_6_) δ (ppm): 3.87 (s, 3H, CH_3_), 6.40 (s, 1H, Ar-H), 6.55 (d, *J*= 9.0 Hz, 1H, Ar-H), 6.99 (d, *J*= 9.0 Hz, 2H, Ar-H), 7.65 (d, *J*= 9.0 Hz, 1H, Ar-H), 7.75(d, *J*= 9.0 Hz, 2H, Ar-H), 9.87 (s, 1H, OH), 13.53 (s, 1H, OH). ^13^C NMR (125 MHz) (DMSO-d_6_) δ (ppm): 55.52 (CH_3_), 103.34, 108.85, 114.41, 123.04, 132.12 (Ar-CH), 134.24, 144.37, 155.56, 161.09, 161.99 (Ar-C).

**4-(4-Nitro-phenylazo)-benzene-1,3-diol (12c)**

Reddish orange crystals from ethanol (89 % yield). mp 192 °C; IR (KBr) cm^-1^: 3340 (OH), 1477 (N=N); ^1^H NMR (400 MHz) (DMSO-d_6_) δ (ppm): 6.38 (S, 1H, Ar-H), 6.52 (d, *J*= 9.0 Hz, 1H, Ar-H), 7.68 (d, *J*= 9.0 Hz, 2H, Ar-H), 7.92 (d, *J*= 9.0 Hz, 2H, Ar-H), 8.07 (d, *J*= 9.0 Hz, 2H, Ar-H), 10.73 (br s, 1H, OH), 12.46 (s, 1H, OH). ^13^C NMR (125 MHz) (DMSO-d_6_) δ (ppm): 103.02, 109.75, 121.55, 129.47, 130.49, 132.85 (Ar-CH), 136.98, 153.18, 157.59, 164.07(Ar-C).

**1-[4-(2,4-Dihydroxy-phenylazo)-phenyl]-ethanone (12d)**

Red crystals from ethanol (84 % yield), mp 220 °C; IR (KBr) cm^-1^: 3350 (OH), 1450(N=N); ^1^H NMR (400 MHz): 2.64 (s, 3H, CH_3_), 6.36 (s, 1H, Ar-H), 6.57 (d, *J*= 9.0 Hz, 1H, Ar-H), 7.66 (d, *J*= 9.0 Hz, 1H, Ar-H), 7.84 (d, *J*= 9.0 Hz, 2H, Ar-H), 8.06 (d, *J*= 9.0 Hz, 2H, Ar-H), 10.43 (br s, 1H, OH), 13.65 (s, 1H, OH). ^13^C NMR (125 MHz) (DMSO-d_6_) δ (ppm): 26.69 (CH_3_), 103.23, 110.25, 121.10, 129.43, 132.84 (Ar-CH), 134.92, 136.86, 152.65, 157.74, 164.28(Ar-C), 196.79 (C=O).

**4-(2,4-Dihydroxy-phenylazo)-benzoic acid (12e)**

Red crystals from ethanol (84 % yield), mp 300 °C; IR (KBr) cm^-1^: 3320 (Br OH), 1510 (N=N); ^1^H NMR (400 MHz): 6.43 (s, 1H, Ar-H), 6.53 (br.s, 1H, Ar-H), 7.64 (d, *J*= 9.0 Hz, 1H, Ar-H), 7.89-8.05 (m, 4H, Ar-H), 11.00 (br s, 1H, OH), 12.02 (br s, 1H, OH), 12.39 (br. s, 1H, OH). ^13^C NMR (125 MHz) (DMSO-d_6_) δ (ppm): 103.14, 104.79, 109.88, 121.65, 122.54, 129.43, 130.67 (Ar-CH), 132.09, 153.32, 154.07, 161.35 (Ar-C), 166.79 (C=O).

**4-(4-Hydroxy-phenylazo)-benzene-1,3-diol (12i & 12j)**

Reddish orange crystals from ethanol (84 % yield), mp 210 °C; IR (KBr) cm^-1^: 3350 (OH), 1515 (N=N); ^1^H NMR (400 MHz): 6.32 (d, *J*= 9.0 Hz, 1H, Ar-H), 6.47 (dd, *J*= 9.0 Hz, 2H, Ar-H), 7.62 (d, *J*= 9.0 Hz, 1H, Ar-H), 7.73 (d, *J*= 9.0 Hz, 2H, Ar-H), 10.15 (br s, 1H, OH), 10.36 (br s, 1H, OH), 12.49 (s, 1H, OH). ^13^C NMR (125 MHz) (DMSO-d_6_) δ (ppm): 103.56, 107.00, 109.71, 114.74, 118.01, 130.67, 131.99 (Ar-CH), 132.63, 152.17, 156.82, 158.85, 163.47 (Ar-C).

##### Synthesis of azo dye based chromene (13a-k)

To a mixture of aryl azo dyes **12a-h** (1 mmol), malononitrile (1 mmol), various benzaldehyde in ethanol (10 mL) and piperidine (0.3 mL) was added under rapid stirring. The reaction was refluxed for 5 h and the solid was filtered and recrystallized from ethanol to afford the pure product.

**2-Amino-4-(4-fluoro-phenyl)-7-hydroxy-6-*p*-tolylazo-4*H*-chromene-3-carbonitrile (13a)**

Brown crystals from ethanol (55 % yield), mp 203 °C; UV/Vis (EtOH): λ_max_= 380 nm; IR (KBr) (cm^-1^): 3410, 3327 (NH_2_), 3203 (OH), 2920 (CH_3_) 2191(CN), 1456 (N=N); ^1^H NMR (400 MHz) (DMSO) δ: 2.41 (s, CH_3_, 3H), 4.83(s, IH, H-4), 6.77 (d, *J*= 9.0 Hz, 1H, Ar-H), 7.04 (br. S, 2H, NH_2_), 7.11 (d, J= 9.0, 2H, Ar-H), 7.23-7.26 (m, 2H, Ar-H), 7.71-7.78 (m, 4H, Ar-H), 7.91 (d, *J*=9.0 Hz, 1H, Ar-H), 11.86 (s, 1H, OH). ^13^C NMR (125 MHz) (DMSO-d_6_) δ (ppm): 21.44 (CH_3_), 35.80 (C-4), 59.61 (C-3), 108.27 (C-8), 112.03 (CN), 115.08 (Ar-CH), 115.22 (C-5), 120.09 (C-4a), 121.86, 129.04 (Ar-CH), 129.09 (C-6), 129.99, 132.55, 134.11 (Ar-CH), 140.18 (Ar-C), 141.73, 147.71 (Ar-CH), 151.63 (C-7), 151.96 (Ar-C), 159.50 (C-8a), 160.70 (C-2), 162.32 (Ar-C). ^19^F NMR (DMSO-d_6_) δ (ppm): -116.24 (Ar-F). MS m\z (%): 400 (M^+^, 20.5) with base peak at 237.

**2-Amino-4-(4-fluoro-phenyl)-7-hydroxy-6-(4-methoxy-phenylazo)-4*H*-chromene-3-carbonitrile (13b)**

Brown crystals from ethanol (40 % yield), mp 272 °C; UV/Vis (EtOH): λ_max_= 386 nm; IR (KBr) (cm^-1^): 3414, 3336 (NH_2_), 3223 (OH), 2185 (CN), 1464 (N=N); ^1^H NMR (400 MHz) (DMSO-d_6_) δ (ppm): 3.84 (s, 3H, CH_3_), 4.79 (s, 1H, H-4), 6.79 (d, *J*= 9.0 Hz, 1H, Ar-H) 7.03 (br. S, 2H, NH_2_), 7.07-7.14 (m, 4H, Ar-H), 7.21-7.25 (m, 2H, Ar-H), 7.74 (d, *J*= 9.0 Hz, 1H, Ar-H), 7.93 (d, *J*= 9.0 Hz, 2H, Ar-H), 12.23 (br. s, 1H, OH). ^13^C NMR (125 MHz) (DMSO-d_6_) δ (ppm): 35.85(C-4), 55.60 (CH_3_), 57.08 (C-3), 108.03 (C-8), 112.11 (CN), 114.59 (Ar-CH), 115.01 (C-5), 115.22 (Ar-CH), 120.08 (C-4a), 124.32 (Ar-CH), 125.46 (C-6), 129.01, 134.46 (Ar-CH), 141.23 (Ar-C), 144.80 (Ar-CH), 151.41 (C-7), 151.83 (C-8a), 159.69 (Ar-C), 161.82 (C-2), 162.10 (Ar-C). ^19^F NMR (DMSO-d_6_) δ (ppm): -116.26 (Ar-F). MS m\z (%): 416 (M^+^, 22.5) with base peak at 186.

**2-Amino-4-(4-fluoro-phenyl)-7-hydroxy-6-(4-nitro-phenylazo)-4*H*-chromene-3-carbonitrile (13c)**

Brown crystals from ethanol (41 % yield), mp 254 °C; UV/Vis (EtOH): λ_max_= 390 nm; IR (KBr) (cm^-1^): 3387,3313 (NH_2_, OH), 2185 (CN), 1458 (N=N); ^1^H NMR (400 MHz) (DMSO-d_6_) δ (ppm): 4.79 (s, 1H, H-4), 6.80 (d, *J*= 9.0 Hz, 1H, Ar-H) 7.06 (s, 2H, NH_2_), 7.13 (t, *J*= 9.0 Hz, 2H, Ar-H), 7.22-7.26 (m, 2H, Ar-H), 7.77 (d, *J*= 9.0 Hz, 1H, Ar-H), 8.06 (dd, *J*= 8.6 Hz, 4H, Ar-H), 12.17 (br. s, 1H, OH). ^13^C NMR (125 MHz) (DMSO-d_6_) δ (ppm): 35.85 (C-4), 57.17 (C-3), 108.69 (C-8), 112.39 (CN), 115.03 (Ar-CH), 115.24 (C-5), 119.95 (C-4a), 122.49, 124.58, 128.99 (Ar-CH), 129.07 (C-6), 129.35, 135.12, 137.86 (Ar-CH), 141.14 (Ar-C), 152.80 (C-7), 153.13, 153.88 (Ar-C), 159.44 (C-8a), 159.72 (C-2), 162.13 (Ar-C). ^19^F NMR (DMSO-d_6_) δ (ppm): -116.19 (Ar-F). MS m\z (%): 431 (M^+^, 20.5) with base peak at 237.

**6-(4-Acetyl-phenylazo)-2-amino-4-(4-fluoro-phenyl)-7-hydroxy-4*H*-chromene-3-carbonitrile (13d)**

Brown crystals from ethanol (52 % yield), mp 265 °C; UV/Vis (EtOH): λ_max_= 390 nm; IR (KBr) (cm^-1^): 3398, 3313 (NH_2_), 3196 (OH), 2187 (CN), 1450 (N=N); ^1^H NMR (400 MHz) (DMSO-d_6_) δ (ppm): 2.62 (s, 3H, CH_3_), 4.79 (s, 1H, H-4), 6.81 (d, *J*= 9.0 Hz, 1H, Ar-H) 7.06 (s, 2H, NH_2_), 7.13 (t, *J*= 9.0 Hz, 2H, Ar-H), 7.22-7.26 (m, 2H, Ar-H), 7.78 (d, *J*= 9.0 Hz, 1H, Ar-H), 8.07 (dd, *J*= 6.8 Hz, 4H, Ar-H), 12.15 (br. s, 1H, OH). ^13^C NMR (125 MHz) (DMSO-d_6_) δ (ppm): 26.85 (CH_3_), 35.85 (C-4), 57.16 (C-3), 108.69 (C-8), 112.41 (CN), 115.04 (Ar-CH), 115.25 (C-5), 119.95 (C-4a), 122.51, 124.44, 128.99 (Ar-CH), 129.07 (C-6), 129.37, 135.15, 137.88, 141.17 (Ar-CH), 152.81 (C-7), 153.17, 153.90 (Ar-C), 159.44 (C-8a), 159.72 (C-2), 162.13 (Ar-C), 197.25 (C=O). ^19^F NMR (DMSO-d_6_) δ (ppm): -116.20 (Ar-F). MS m\z (%): 428 (M^+^, 30.5 ) with base peak at 109.

**4-[2-Amino-3-cyano-4-(2-fluoro-phenyl)-7-hydroxy-4*H*-chromen-6-ylazo]-benzoic acid (13e)**

Brown crystals from ethanol (52 % yield), mp 243 °C; UV/Vis (DMF): λ_max_= 388 nm; IR (KBr) (cm^-1^): 3420-3385 (NH_2_), 2230 (CN), 1645 (C=O), 1520 (N=N); ^1^H NMR (400 MHz) (DMSO) δ: 5.05 (s, IH, H-4), 6.78 (d, *J*= 9.0 Hz, 1H, Ar-H), 7.10 (br. s, 2H, NH_2_), 7.13-7.17 (m, 3H, Ar-H), 7.24-7.27 (m, 1H, Ar-H), 7.78 (d, *J*= 9.0 Hz, 1H, Ar-H), 7.95 (d, *J*=9.0 Hz, 2H, Ar-H), 8.03 (d, *J*=9.0 Hz, 2H, Ar-H), 10.92 (br. S, 1H, COOH).^ 13^C NMR (125 MHz) (DMSO-d_6_) δ (ppm): 31.35 (C-4), 55.95 (C-3), 108.50 (C-8), 111.69 (CN), 115.85 (Ar-CH), 116.06 (C-5), 120.58 (C-4a), 122.46, 124.85, 129.16, 130.35, 130.58 (Ar-CH), 132.16 (C-6), 135.42 (Ar-CH), 152.61 (C-7), 153.30, 154.32 (Ar-C), 159.16 (C-8a), 160.25 (C-2), 161.69 (Ar-C), 168.61 (C=O).^19^F NMR (DMSO-d_6_) δ (ppm): -119.76 (Ar-F). MS m\z (%): 430 (M^+^, 20.5) with base peak at 237.

**4-[2-Amino-3-cyano-4-(2-fluoro-phenyl)-7-hydroxy-4*H*-chromen-6-ylazo]-benzenesulfonic acid (13f)**

Brown crystals from ethanol (59 % yield), mp 290 °C; UV/Vis (DMF): λ_max _= 390 nm; IR (KBr) (cm^-1^): 3450- 3425 (NH_2_), 2220(CN), 1495 (N=N); ^1^H NMR (400 MHz) (DMSO) δ: 5.04(s, IH, H-4), 6.80 (d, *J*= 9.0 Hz, 1H, Ar-H), 7.08 (br. s, 2H, NH_2_), 7.11-7.16 (m, 3H, Ar-H), 7.22-7.27 (m, 1H, Ar-H), 7.73 (d, *J*= 9.0 Hz, 3H, Ar-H), 7.78 (d, *J*= 9.0 Hz, 1H, Ar-H), 7.92 (d, *J*=9.0 Hz, 2H, Ar-H), 11.77 (br. s, 1H, OH). ^13^C NMR (125 MHz) (DMSO-d_6_) δ (ppm): 31.31 (C-4), 56.01 (C-3), 107.73 (Ar-CH), 108.65 (C-8), 111.55 (CN), 115.86 (Ar-CH), 116.08 (C-5), 120.45 (C-4a), 122.56, 125.01, 127.08, 129.12, 130.34 (Ar-CH), 131.97 (C-6), 135.25 (Ar-CH), 150.69 (Ar-C), 151.04 (C-7), 153.04, 153.65 (Ar-C), 159.15 (C-8a), 160.25 (C-2), 161.59 (Ar-C). ^19^F NMR (DMSO-d_6_) δ (ppm): -119.77 (Ar-F). MS m\z (%): 466 (M^+^, 20.5) with base peak at 237.

**4-[2-Amino-4-(4-tert-butyl-phenyl)-3-cyano-7-hydroxy-4*H*-chromen-6-ylazo]-benzenesulfonic acid (13g)**

Brown crystals from ethanol (67 % yield), mp 330 °C; UV/Vis (DMF): λ_max _= 386 nm; IR (KBr) (cm^-1^): 3430-3400 (NH_2_), 2225 (CN), 1510 (N=N); ^1^H NMR (400 MHz) (DMSO) δ: 1.23 (s, 9H, CH_3_), 4.73 (s, IH, H-4), 6.81 (d, *J*= 9.0 Hz, 1H, Ar-H), 7.05 (br. s, 2H, NH_2_), 7.11(d, *J*=9.0 Hz, 2H, Ar-H), 7.32 (d, *J*=9.0 Hz, 2H, Ar-H), 7.73-7.79 (m, 3H, Ar-H), 7.93 (d, J=9.0 Hz, 2H, Ar-H). ^13^C NMR (125 MHz) (DMSO-d_6_) δ (ppm): 31.53 (CH_3_), 34.53 (C-CH_3_), 36.37 (C-4), 57.61 (C-3), 108.74 (C-8), 113.13 (CN & C-5), 120.73 (C-4a), 122.46 (Ar-CH), 124.95 (C-6), 125.67, 127.02, 127.15, 135.23, 142.42 (Ar-CH), 149.30, 150.71 (Ar-C), 150.92 (C-7), 152.76 (Ar-C), 153.32 (C-8a), 160.08 (C-2). MS m\z (%): 504 (M^+^, 20.5) with base peak at 237.

**4-[2-Amino-4-(4-butyl-phenyl)-3-cyano-7-hydroxy-4*H*-chromen-6-ylazo]-benzenesulfonic acid (13h)**

Brown crystals from ethanol (63 % yield), mp 275 °C; UV/Vis (DMF): λ_max _= 385 nm; IR (KBr) (cm^-1^): 3450, 3425 (NH_2_), 2210 (CN), 1510 (N=N); ^1^H NMR (400 MHz) (DMSO) δ: 0.88 (t, *J*=6.0 Hz, 3H, CH_3_), 1.26-1.31 (m, 2H, CH_2_), 1.47-1.55 (m, 2H, CH_2_), 2.49 (br. s, 2H, CH_2_), 4.73 (s, IH, H-4), 6.81 (d, *J*= 9.0 Hz, 1H, Ar-H), 7.05 (br. s, 2H, NH_2_), 7.07-7.13 (m, 4H, Ar-H), 7.73-7.80 (m, 3H, Ar-H), 7.93 (d, *J*=9.0 Hz, 2H, Ar-H), 11.93 (br. S, 1H, OH). ^13^C NMR (125 MHz) (DMSO-d_6_) δ (ppm): 14.14 (CH3), 22.23 (CH2), 33.52 (CH_2_), 34.85 (CH_2_), 36.51 (C-4), 57.65 (C-3), 108.73 (C-8), 113.12 (CN & C-5), 120.67 (C-4a), 122.43 (Ar-CH), 125.10 (C-6), 127.01, 127.40, 128.74, 135.19, 141.08 (Ar-CH), 142.66 (Ar-C), 150.86 (C-7), 152.72 (Ar-C), 153.31 (C-8a), 160.03 (C-2). MS m\z (%): 504 (M^+^, 20.5) with base peak at 237.

**2-Amino-4-(4-tert-butyl-phenyl)-7-hydroxy-6-(4-hydroxy-phenylazo)-4*H*-chromene-3-carbonitrile (13i)**

Brown crystals from ethanol (79 % yield), mp 255 °C; UV/Vis (DMF): λ_max _= 387 nm; IR (KBr) (cm^-1^): 3350- 3300 (NH_2_), 2215 (CN), 1520 (N=N); ^1^H NMR (400 MHz) (DMSO) δ: 1.23 (s, 9H, CH_3_), 4.72 (s, IH, H-4), 6.75 (d, *J*= 9.0 Hz, 1H, Ar-H), 6.87 (d, *J*= 9.0 Hz, 2H, Ar-H), 7.01 (br. s, 2H, NH_2_), 7.11 (d, *J*=9.0 Hz, 2H, Ar-H), 7.31 (d, *J*=9.0 Hz, 2H, Ar-H), 7.70 (d, *J*= 9.0 Hz, 1H, Ar-H), 7.80 (d, *J*=9.0 Hz, 2H, Ar-H). 31.52 (CH_3_), 34.50 (C-CH_3_), 36.40 (C-4), 57.63 (C-3), 107.81 (C-8), 113.03 (CN), 116.58 (Ar-CH & C-5), 120.90 (C-4a), 124.96 (Ar-CH), 125.61 (Ar-CH & C-6), 127.12, 135.03, 142.61 (Ar-CH), 143.74 (Ar-C), 149.19 (C-7), 151.61 (Ar-C), 152.63 (C-8a), 160.28 (C-2), 162.13 (Ar-C). MS m\z (%): 440 (M^+^, 20.5) with base peak at 237.

**2-Amino-4-(2-fluoro-phenyl)-7-hydroxy-6-(4-hydroxy-phenylazo)-4*H*-chromene-3-carbonitrile (13j)**

Red crystals from ethanol (62 % yield), mp 260 °C; UV/Vis (DMF): λ_max_= 387 nm; IR (KBr) (cm^-1^): 3330-3310 (NH_2_), 2215 (CN), 1520 (N=N); ^1^H NMR (400 MHz) (DMSO) δ: 5.04 (s, IH, H-4), 6.76 (d, *J*= 9.0 Hz, 1H, Ar-H), 6.88 (d, *J*= 9.0 Hz, 2H, Ar-H), 7.05 (br. s, 2H, NH_2_), 7.11-7.17 (m, 3H, Ar-H), 7.22-7.27 (m, 1H, Ar-H), 7.71 (d, *J*= 9.0 Hz, 2H, Ar-H), 7.81 (d, *J*=9.0 Hz, 2H, Ar-H). ^13^C NMR (125 MHz) (DMSO-d_6_) δ (ppm): 31.23 (C-4), 56.00 (C-3), 108.03 (C-8), 111.28 (CN), 115.79 (Ar-CH), 116.00 (C-5), 116.48 (Ar-CH), 120.51 (C-4a), 124.96, 125.04, 126.05 (Ar-CH), 129.06 (Ar-C), 130.29 (Ar-CH), 132.07 (C-6), 134.79, 144.00 (Ar-CH), 151.88 (C-7), 152.35 (Ar-C), 159.08 (C-8a), 160.35 (C-2), 161.61 (Ar-C). ^19^F NMR (DMSO-d_6_) δ (ppm): -119.70 (Ar-F). MS m\z (%): 402 (M^+^, 20.5) with base peak at 237.

**2-Amino-4-(2-fluoro-phenyl)-7-hydroxy-6-(3-hydroxy-phenylazo)-4*H*-chromene-3-carbonitrile (13k)**

Reddish brown crystals from ethanol (68 % yield), mp 280 °C; UV/Vis (DMF): λ_max_= 388 nm IR (KBr) (cm^-1^): 3475 (NH_2_), 2220 (CN), 1515 (N=N); ^1^H NMR (400 MHz) (DMSO) δ: 5.04(s, IH, H-4), 6.81(d, J= 9.0 Hz, 1H, Ar-H), 6.93(d, J= 9.0 Hz, 1H, Ar-H), 7.10 (br. s, 2H, NH_2_), 7.13-7.17 (m, 3H, Ar-H), 7.25-7.27 (m, 1H, Ar-H), 7.31-7.35 (m, 2H, Ar-H), 7.41 (s, 1H, Ar-H), 7.78 (d, J = 9.0 Hz, 1H, Ar-H), 9.81 (br. S, 1H, OH), 12.41 (br. S, 1H, OH). ^13^C NMR (125 MHz) (DMSO-d_6_) δ (ppm): 31.23 (C-4), 55.94 (C-3), 107.73 (Ar-CH), 108.60 (C-8), 111.42 (CN), 115.39, 115.80 (Ar-CH), 116.02 (C-5), 118.78 (Ar-CH), 120.35 (C-4a), 124.97, 126.97 (Ar-CH), 129.04 (Ar-C), 130.50 (Ar-CH), 131.85 (C-6), 134.80 (Ar-CH), 152.16 (C-7), 152.97, 158.63 (Ar-C), 159.14 (C-8a), 160.18 (C-2), 161.51 (Ar-C). ^19^F NMR (DMSO-d_6_) δ (ppm): -119.74 (Ar-F). MS m\z (%): 402 (M^+^, 20.5) with base peak at 237.

## Biological Studies

### Antimicrobial screening

The microorganism inoculums were uniformly spread using sterile cotton swabs on a sterile Petri dish malt extract agar (for fungi) and nutrient agar (for bacteria). One hundred cubic millimeters of each sample was added to each well (10 mm-diameter holes were cut in the agar gel, 20 mm apart from one another). The systems were incubated for 24-48 h at 37 °C (for bacteria) and at 28 °C (for fungi). After incubation, the microorganism growth was observed. Inhibition zones of the bacterial and fungal growth were measured in millimeters. Tests were performed in triplicate (Cappuccino and Sherman, 1999[12]; Vanden Berghe and Vlientinck, 1991[62]). 

### Cytotoxic screening

Human colon carcinoma (HCT-116), human hepatocellular carcinoma (HEPG-2), adenocarcinomic human alveolar basal epithelial cell (A-549), and human breast adenocarcinoma (MCF-7) cell lines were obtained from the American Type Culture Collection (ATCC, Rockville, MD). The cells were grown on RPMI-1640 medium supplemented with 10 % inactivated fetal calf serum and 50 µg/ml gentamycin. The cells were maintained at 37 °C in a humidified atmosphere with 5 % CO_2_ and were subcultured two to three times a week. Potential cytotoxicity of the compounds was evaluated on tumor cells using the method of Gangadevi and Muthumary (2007[21]) (Klancnik et al., 2010[36]). The cells were grown as monolayers in growth RPMI-1640. The monolayers of 104 cells adhered at the bottom of the wells in a 96-well microtiter plate incubated for 24 h at 37 °C in a humidified incubator with 5 % CO_2_. The monolayers were then washed with sterile phosphate buffered saline (0.01 M pH 7.2) and simultaneously the cells were treated with 100 µL from different dilutions of tested sample in fresh maintenance medium and incubated at 37 °C. A control of untreated cells was made in the absence of tested sample. Positive controls containing doxorubicin were also tested as a reference drug for comparison. Six wells were used for each concentration of the test sample. Every 24 hours an observation under the inverted microscope was made. The number of the surviving cells was determined by staining the cells with crystal violet (Mosmann, 1983[42]; Gangadevi and Muthumary, 2007[21]) followed by cell lysing using 33 % glacial acetic acid and reading the absorbance at 590 nm using microplate reader (SunRise, TECAN, Inc, USA) after well mixing. The absorbance values from untreated cells were considered as 100 % proliferation. The number of viable cells was determined using microplate reader as previously mentioned before and the percentage of viability was calculated as [1-(ODt/ODc)] x 100 % where ODt is the mean optical density of wells treated with the tested sample and ODc is the mean optical density of untreated cells. The relation between surviving cells and drug concentration was plotted to get the survival curve of each tumor cell line after treatment with the specified compound. The 50 % inhibitory concentration (IC_50_), the concentration required to cause toxic effects in 50 % of intact cells, was estimated from graphic plots.

#### Caspase activity assay

To measure caspase-3 and -7 activities, a luminescent Caspase-Glo 3/7 assay was performed. HCT116 and MCF-7 cells were seeded onto black 96-well plates at a density of 3,500 cells/well. After 24 h the compounds that were tested (**4a, 4b**, and **4c**) and a reference (Doxorubicin) drug at IC_50_ concentration was added. After 48 h a Caspase-Glo 3/7 Assay (Promega) was performed according to the manufacturer's instructions. After adding 100 μL of Caspase 3/7 Glo Reagent, cells were incubated for 2.5 h at room temperature. The luminescence was measured using a multi-plate reader (Synergy 4, BioTek) with an integration time of 1 second per well. The values are the means ± SD of three sets of experiments.

#### In vitro inhibitory activity screening of E. coli DNA gyrase

Gyrase B and ParE monomers have only modest ATPase activity which is enhanced in the case of the holo enzymes and stimulated further by DNA. The phosphate released following conversion of ATP into ADP can be detected by the addition of malachite green solution and measured by monitoring the increase in absorbance at 600 nm. Topoisomerases were purchased from Inspiral is Ltd (Norwich, United Kingdom). For the *Escherichia coli *DNA gyrase ATPase assay, the final assay composition was 10 nM DNA gyrase (a complex of two GyrA and two GyrB subunits, the A2B2 complex) 3 mM Tris, pH 7.5, 24 mM KCl, 2 mM MgCl_2_, 6.5 % (w/v) glycerol, 0.01 mg/ml bovine serum albumin (BSA), 2 mM DTT (dithiothreitol), 9 mM ATP, and 5 % DMSO solution containing the compounds). For *E. coli* topoisomerase IV ATPase assay, the final assay composition was 10 nM topoisomerase IV (a complex consisting of two ParC and two ParE subunits), 40 mM HEPES-KOH, pH 7.6, 100 mM Potassium Glutamate, 25 mM Magnesium Acetate, 10 g/ml single-stranded DNA, 0.2 mg/ml BSA, 10 mM DTT, 0.5 mM ATP, and 5 % DMSO solution containing the compounds. The reactions were started by the addition of the ATP, and the reaction mixtures were allowed to incubate at 30 °C for 60 mins. Reactions were stopped by adding Malachite green solution (0.034 % malachite green, 10 mM ammonium molybdate, 1M HCl, 3.4 % Ethanol, 0.01 % Tween 20). Color was allowed to develop for 5 min, and the absorbance at 600 nm was measured spectrophotometrically. The half-maximum (50 %) inhibitory concentration (IC_50_) values were determined from the absorbance readings using no-compound and no-enzyme controls. The values reported are the averages of at least four independent experiments.

#### Acute toxicity

*In vitro *experiments were carried out at the University of Prince Edward Island and approved by the Animal Care and Biosafety Committees, which adheres to the guidelines of the Canadian Council on Animal Care (protocol #14-041 and 15-028). Mixed neocortical cultures, containing both neurons and glia, were prepared from fetal rats at 17-18 days gestation. Briefly, embryonic tissue was extracted from untimed pregnant Sprague-Dawley rats by caesarean section and transferred to ice-cold Hanks Balanced Salt Solution (Gibco). Cortical brain tissue was carefully isolated by fine dissection using aseptic techniques. Cortices were minced in cold HBSS using a sterile razor blade prior to digestion with 0.0125 % trypsin. Dissociated cells were plated in poly-L-lysine (1 mg/ml, Sigma Aldrich) coated 96 well plates at a seeding density of 50000 cells/well in warm (37 °C) Dulbecco's Modified Eagle Medium (Gibco) containing 10 % iron-supplemented bovine calf serum (Hyclone) and 1 % antibiotic/antimycotic (Gibco). Cultures were allowed to adhere overnight in a humidified 37 °C incubator having 5 % CO_2_ and atmospheric oxygen. The following day, the media was replaced with warmed Neurobasal A Medium (Gibco) containing 0.5 mM L-glutamine (Gibco) and supplemented with 1 % B27, N_2_ and antibiotic/antimycotic (Gibco). Half-volume media changes were made every 4 days thereafter with experiments being carried out on 14-16 day old cultures. After 14 days in culture, the medium was replaced with deoxygenated unsupplemented glucose-free Neurobasal A containing either 10 or 100 µM of the novel compounds T3, T4, T5, T8, T9, T11, or T12. Control wells contained glucose-free medium plus vehicle - either 0.1 % ethanol or 0.1 % DMSO. Cultures were transferred to a humidified 37 °C incubator having 5 % CO_2_, 1.1 % O_2_ and the balance N_2_ for a period of 24 hours. Following OGD, an equal volume of normal supplemented Neurobasal A containing identical drug concentrations was added and cultures were transferred to normoxic conditions (5 % CO_2_ and atmospheric oxygen) for an additional 24 h. Cell viability was assessed by measuring LDH release from damaged cells. Control cultures were prepared in separate plates having supplemented Neurobasal A and being maintained in 5 % CO_2_ and atmospheric oxygen for the duration of the experiment. Toxicity was assessed in wells treated with 10 µM of each of the test compounds listed above. After 24 h an equal volume of culture media as added to each well to account for handling effects on experimental outcomes. Toxicity was assessed after an additional 24 h in culture by measuring LDH release from treated cells. At the end of each experiment, cell injury was assessed by measuring lactate dehydrogenase (LDH) released from damaged cells into the culture medium (Cytoscan LDH Assay, G Biosciences). LDH measurements represent the average of 7 replicate treatments/dose. The effect of OGD and drug treatments on LDH was expressed as a percent of LDH release compared to OGD and vehicle groups. Treatments were compared using a one-way ANOVA followed by Bonferroni post-hoc analysis. Differences were considered significant if p < 0.05. 

### Qualitative chelation analysis

Spectroscopic analysis of chelation property of novel compounds were identified using chemical reaction with ferric chloride. It was done by dissolving of 0.5 gm of compound **4c** as a representative example in 5 ml of methanol and 0.5 ml of diluted HCl. The solution was then mixed with equivalent amount of ferric chloride reagent with stirring for 1 h. The absorbance for reaction mixture and single compound was measured using UV-Visible Spectrophotometer Evolution 201 (Thermoscientific).

### Computational analysis

#### 2D QSAR-based descriptors analysis

All molecular descriptors supplied by the program MOE were computed for QSAR analysis. QSAR-Contingency (Molecular Operating Environment (MOE), Chemical Computing Group, 2012[13]; Hogg et al., 1993[25]), a statistical application in MOE, was used for the selection of relevant descriptors. PLS analysis was performed to determine the relationship between these 2D molecular descriptors and biological activity of the compounds. The predictive ability of the model was determined by classical leave one out (LOO) and leave one pair out cross validation procedures.

#### Docking studies

Docking was carried out to the active compounds **4a-c, **and **7c** with the selective pharmacologically important drug targets that overexpressed in *breast and colon cancers* using docking module implemented in MOE software (Molecular Operating Environment (MOE), Chemical Computing Group, 2012[13]). The drug targets namely epidermal growth factor receptor (EGFR) (PDB id: 4HJO), and Caspase 3 (PDB id: 2XYP) were retrieved from the protein databank (Berman et al., 2000[8]). Initially all the structures were protonated with addition of polar hydrogens followed by energy minimization with MMFF94x force field in order to get stabilized conformer of the protein. As per the literature the inhibitor binding sites were identified and highlighted with site finder module implemented in MOE software and docking was carried out with default parameters.

## Results and Discussion

### Chemistry

#### Synthesis of chromene compounds

**Figure 2:**
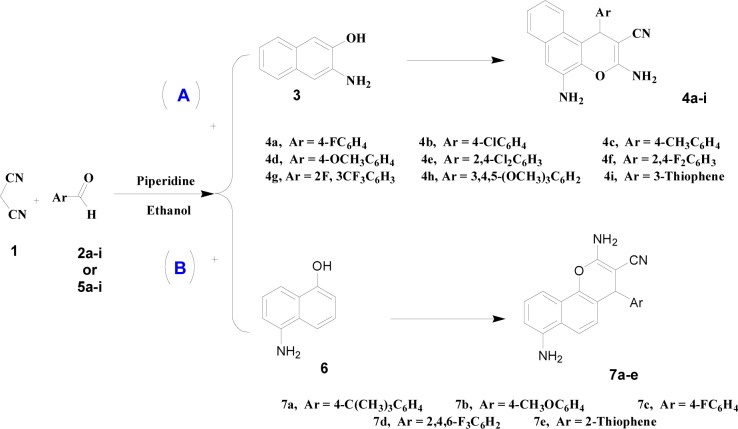
Synthesis of two series of chromene derivatives

The new compounds have been synthesized using Knoevenagel condensation followed by Michael addition adducts (Widelski et al., 2009[66]). Two new families of chromene compounds were synthesized via multi-component reactions (MCRs) in order to examine their biological structure activity relationship (Figure 2(Fig. 2)). The first family has been obtained via reaction of malononitrile **1** and various substituents of aromatic aldehydes **2a-i **with 3-amino-2-naphthol **3** in the presence of piperidine, route A. The solutions were refluxed for 2 h and afforded 3,5-diamino-4-aryl-1H-benzo chromene-2-carbonitrile compounds **4a-i** in 60-77 % yield. The second series of chromene derivatives has been prepared using the same methodology. Route B illustrates the reaction of malononitrile **1** and various aldehydes **5a-e** with 5-amino-1-naphthol **6 **to produce 2,7-Diamino-4-aryl-4*H*-benzo[*h*]chromene-3-carbonitrile **7a-e**.

The structures of the obtained compounds **4a-i** and **7a-e** were characterized using FT-IR and NMR spectroscopic techniques. For instance, the FT-IR spectroscopy showed a characteristic absorption band between 2185 and 2210 cm^-1^ for the CN group, while the NH_2_ stretches were in the range of 3320-3490 cm^-1^. The ^1^H NMR spectra of chromene compounds **4a-i** in DMSO-d_6_ showed signals at 5.09-5.67 ppm for 4-H pyran, while the singlets at 5.43-5.66 ppm are corresponding to NH_2_-10. The NH_2_-2 has a signal overlapped with the aromatic protons in the range of 6.84-7.78 ppm for all chromene derivatives except for compounds **4a** and **4g**, which had the protons of NH_2_-2 as a singlet at δ 6.85 and 7.05 ppm, respectively. The ^13^C NMR spectra showed a signal at δ 32.96-39.08 ppm corresponding to C-4, while C-3 appeared in the range of 56.06-58.66 ppm. The C-2 resonance showed signal at 159.71-160.89 ppm and the CN resonance was in the range of 120.17-122.61 ppm. It is also important to mention that chromene molecule **7c** has been reported previously in literature (Yadav et al., 2007[67]), while their counterparts **7a, b, d** and **e** have not been reported yet. 

#### Synthesis of chromene-based azo dyes 

**Figure 3:**
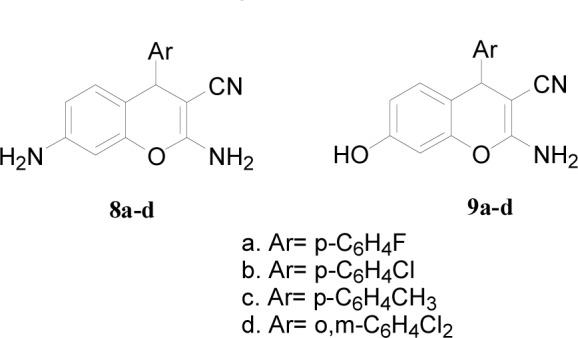
2,7-amino-4-aryl-3-cyano-4H-chromene (8a-d) and 2-amino-4-aryl-7-hydroxy-3-cyano-4H-chromene (9a-d)series

**Figure 4:**
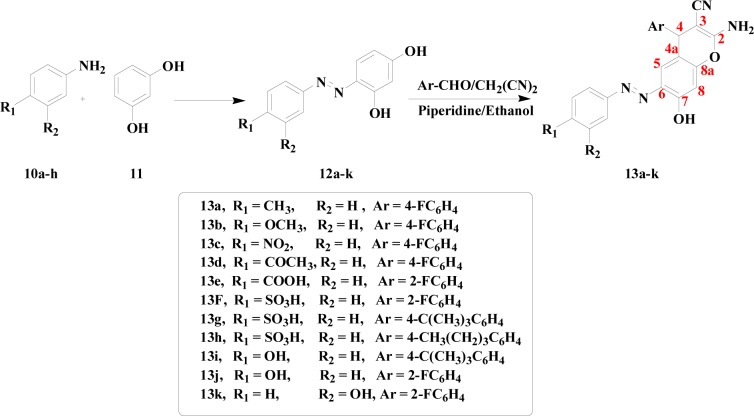
Synthesis of azo chromene dyes 13a-k

Two different series of chromene compounds have also been prepared using MCR in order to use them as precursors for building azo dye molecules. Compounds **8a-d** and **9a-d** were prepared according to a previous report (Figure 3(Fig. 3)) (Zheng et al., 2010[68]). Upon the synthesis of these two series, they been employed as amines or as couplers for further reaction to prepare their azo chromophore counterparts. However, this methodology was not successful since the analysis of the obtaining materials showed disappearance of the cyano group which suggested two possibilities; either the formation of the carboxylic acid via hydrolysis reaction of the cyano group or the pyran ring had been opened during the reaction. To overcome this problem, another attempt had been achieved to prepare the chromene azo dye via different approach. The new synthetic approach allowed for successful isolation of novel series of azo chromophores via two steps' methodology. The first step included the synthesis of arylazo dyes **12a-k** through reactions of diazonium salts of aromatic amines with resorcinol (Afifi, 2003[3]). These compounds were then employed for further reactions with malononitrile and derivatives of benzaldehyde to obtain the first example of azo dyes containing chromene moieties **13a-k** (Figure 4(Fig. 4)). 

Compounds **13a-k** represent a novel class of azo dyes. The structures of these new dyes were confirmed using the spectroscopic analytical techniques. In the IR spectra, the absorptions at 2185-2191 cm^-1^ were assigned to the CN functionality, while the absorptions at 1450-1464 cm^-1^ were assigned to (N=N) moiety. The ^1^H NMR spectra showed the presence of signals for H-4, appearing as a singlet in the range of δ 4.72-5.05 ppm, while the NH_2_-2 protons appeared in the range of 7.01-7.10 ppm. The rest of the aromatic protons of the chromene moieties and the arene rings appeared downfield in the range of 7.07-8.07 ppm. The ^13^C NMR spectra showed the resonance of C-4 at 31.23-36.51 ppm, while the CN resonance at 111.28-113.13 ppm and C-2 resonance at 159.72-161.82 ppm. And finally, the ^19^F NMR spectra showed the presence of the florin signals between -116.19 and -119.77 ppm. 

#### UV-vis study

A UV-vis study of azo dyes **12a-k** and their azo chromene counterparts' **13a-k** was performed in DMF in order to explore their λ_max_ values. The obtained data showed that the formation of the chromene azo derivatives did not alter the λ_max_ values of their azo precursors due to the non-conjugated system of the chromene moieties. All azo compounds and their azo chromene analogues displayed λ_max_ in the range of 390-404 nm. 

### Prediction of activity spectra for substances (PASS) 

The Prediction of Activity Spectra for Substances (PASS) has been utilized in order to predict the spectrum of activity (Stepanchikova et al., 2003[59]). In this study, we explored the biological potential of selected compounds to prioritize them for further *in vitro* studies. The PASS is an *in silico* tool used for predicting biological activity spectra for natural and synthetic substances, which is based on the structure-activity relationships knowledgebase for more than 260,000 compounds with known biological activities including drugs, drug candidates, pharmaceutical leads and toxic compounds (Patil et al., 2015[46]). The PASS predicts the tentative biological potential of the compound based on its structure and reveals the predicted activities as the probability of activity (P_a_) and inactivity (P_i_). The higher the P_a_ value, the lower the predicted probability of obtaining false positives in biological testing. The PASS analysis revealed that target compounds have high activity scores as apoptotic agonists with P_a_ values in the 0.851-0.721 range. This prediction agrees with previous published work (Elinson et al., 2010[20]; Sondhi et al., 2010[58]; Thareja et al., 2010[60]; Trinchieri, 2015[61]). The obtained *in silico* findings were further validated *in vitro* by different biological analyses including the reported antimicrobial activity and cytotoxic screening as described below. 

### Biological screening

#### Antimicrobial screening

**Figure 5:**
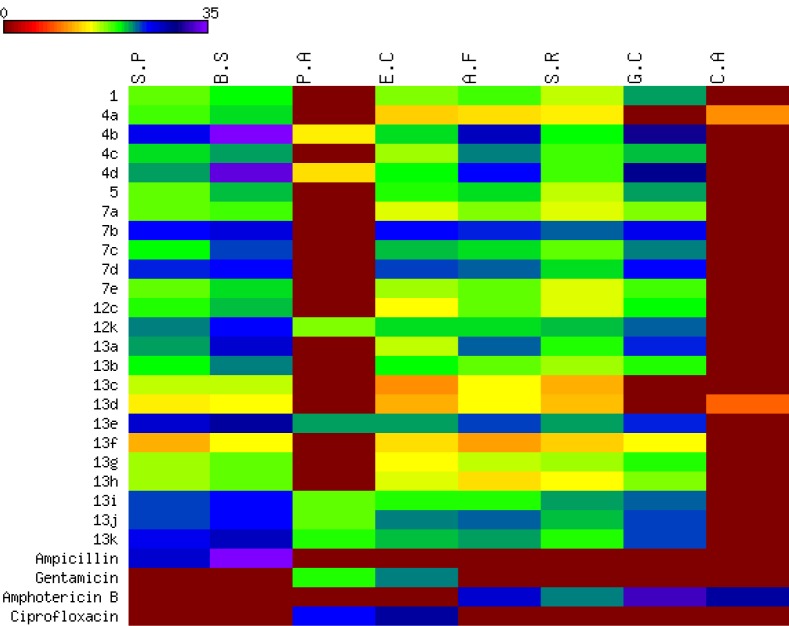
Heat map of inhibition zone data for the tested compounds against bacterial and fungal strains. The values are color-coded and color bars mark the matrix positions of compounds in a particular bacteria type.

**Figure 6:**
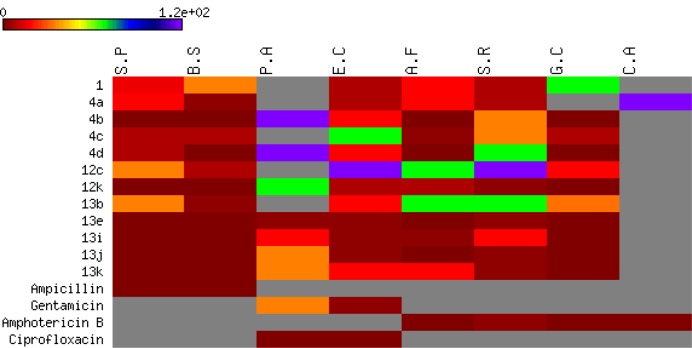
Heat map of MIC data for the tested active compounds against bacterial and fungal strains. The values are color-coded and color bars mark the matrix positions of compounds in a particular bacteria type.

All the newly synthesized compounds described in this report were screened for both antibacterial and antifungal activity via the reported agar diffusion well method (Vanden Berghe and Vlietinck, 1991[62]). The inhibition zones and minimum inhibitory concentrations (MIC) were determined by the serial dilution method (Cappuccino and Sherman, 1999[12]). The antimicrobial activity of the synthesized compounds was tested against a broad panel of microorganisms including two Gram-positive bacteria: *Streptococcus pneumoniae* (RCMB 010010) and *Bacillus subtilis* (RCMB 010067), two Gram-negative bacteria: *Pseudomonas aeruginosa* (RCMB 010043) and *Escherichia coli* (RCMB 010052), and four fungi: *Aspergillus fumigatus *(RCMB 02568), *Syncephalastrum racemosum* (RCMB 05922*), Geotricum candidum* (RCMB 05097), and *Candida albicans* (RCMB 05036). Ampicillin, ciprofloxacin, gentamicin and amphotericin B were used as positive control drugs (Atta-ur-Rahman and Thomsen, 2001[7]; Smania et al., 1999[57]). The observed inhibition zone (IZ) and minimum inhibitory concentrations (MIC) of the tested compounds and the reference drugs are given in Tables 1(Tab. 1) and 2(Tab. 2) and presented in Figures 5(Fig. 5) and 6(Fig. 6). It appeared that most of the tested compounds showed appreciable bacterial and fungal inhibition compared to reference drugs. In general, these compounds displayed mostly inhibitory activity against Gm+ve bacteria resulting in an IZ range from 11 to 33 mm. While in the case of Gm-ve bacteria, they had weak activity. In addition, certain strains of fungi were sensitive to tested compounds with an IZ range from 12-25 except *Candida albicans*. Among the synthesized compounds, **4b **and** 4d **derivatives were found to be more effective against Gm+ve and fungi with an IZ range from 18 to 30 mm and MIC from 0.007 to 0.49 µg/ml (more potent than positive controls). While compounds **7b **and **7d** were also found to be more active against Gm+ve, Gm-ve bacteria, and fungi with an IZ ranging from 18-23 mm. In the case of antibacterial activity against Gm+ve bacteria, most of the compounds were found to be comparable to the positive controls, having an MIC ranging from 3.9 to 0.49 µg/ml. 

The chromene azo derivatives showed moderate inhibitory activity against all tested microorganisms especially compound **13e**, **13j**, and **13j **with MIC values of 0.007, 0.12, 0.49, and 3.9 µg/ml. Generally, the investigation of antimicrobial activity of the novel derivatives showed, in part, that some of them were more potent compared to the positive controls used against Gm+ve bacteria and certain strains of fungi.

#### Cytotoxic screening

**Figure 7:**
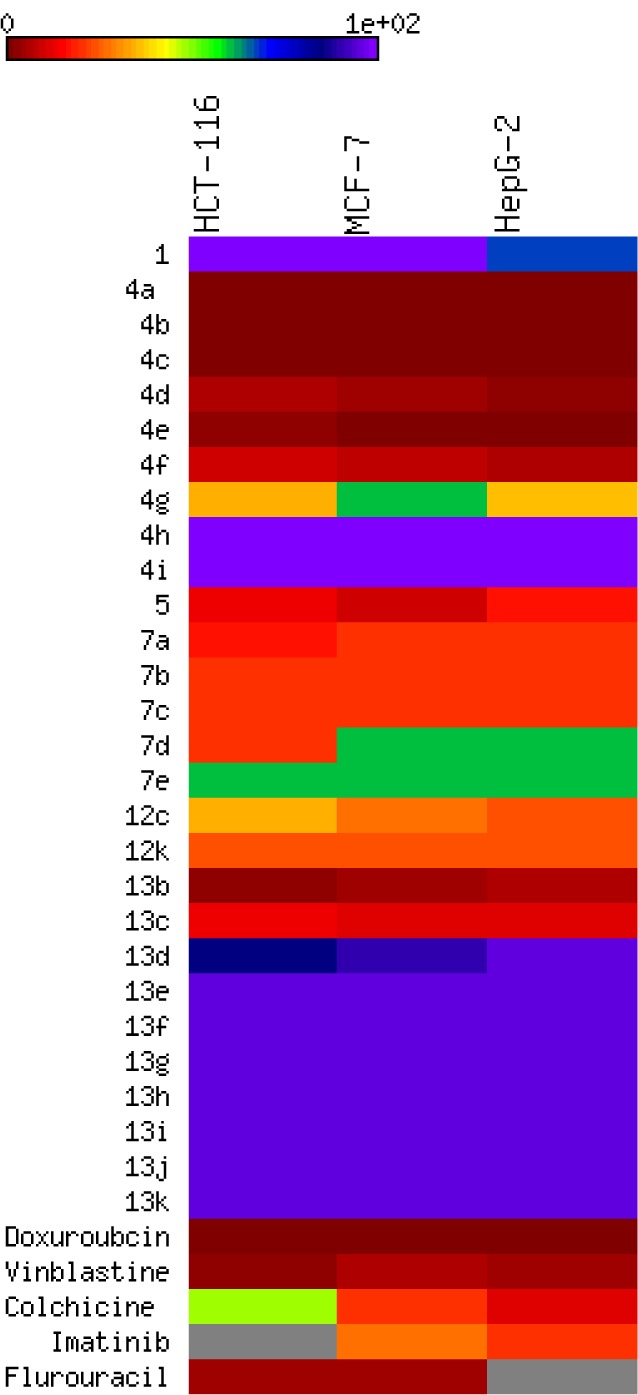
*In vitro* cytotoxic activity. The IC_50_ values of target compounds compared to reference drug are color-coded according to the following scheme: green 0.5-10, black 11-14, and red 15-24. Color bars mark the matrix positions of compounds in a particular cell line.

The *in vitro* cytotoxic activity was performed by MTT assay (Mosmann, 1983[42]; Alley et al., 1988[6]) against three human carcinoma cell lines: Human colon carcinoma (HCT-116), human breast adenocarcinoma (MCF-7), and human hepatocellular carcinoma (HEPG-2). Doxorubicin was used as a positive control with reported high cytotoxic activity. The inhibitory effects of tested compounds on the growth of the three cell lines are shown in Tables 3(Tab. 3) and 4(Tab. 4) and Figure 7(Fig. 7). All compounds showed potent or comparable cytotoxicity to the positive control. From the observed data, it was noted that 3-amino-2-naphthol starting material showed weak antiproliferative activity by 60 to 230 µg/mL. The activity is dramatically changed by the formation of benzochromene derivatives **4a-g** with IC_50_ ranging from 0.3 to 50 µg/mL. Three derivatives **4a-c** exhibited equally potent activity against three cell lines. Increasing the size of the substituents on the aromatic ring led to a loss of activity as in **4h-i**. While in the case of the starting material 5-amino-1-naphthol, the activity was moderate with an IC_50_ ranging from 9 to 15 µg/mL. Hence, the benzochromene derivatives **7a-e** showed moderate inhibitory activity towards all cell lines ranging from 11.9 to 61.9 µg/mL. One derivative displayed potent activity compared to the reference drug with an IC_50_ from 0.7 to 2 µg/mL against all target cell lines. In the case of azo chromene derivatives **13b-k**, they showed weak to moderate activity compared to the reference drugs with an IC_50_ ranging from 10.4 to 100 µg/mL. Of this group, only compound **13b** displayed potent (IC_50_ form 2.41 to 5.4 µg/mL) activity. Thus, most of synthesized compounds showed promising antiproliferative activity compared to previously reported work (Yadav et al., 2007[67]; Zheng et al., 2010[68]; El-Agrody et al., 2000[19]; Sabry et al., 2011[50]), but would need to be improved for further optimization.

#### Caspase activity assay

**Figure 8:**
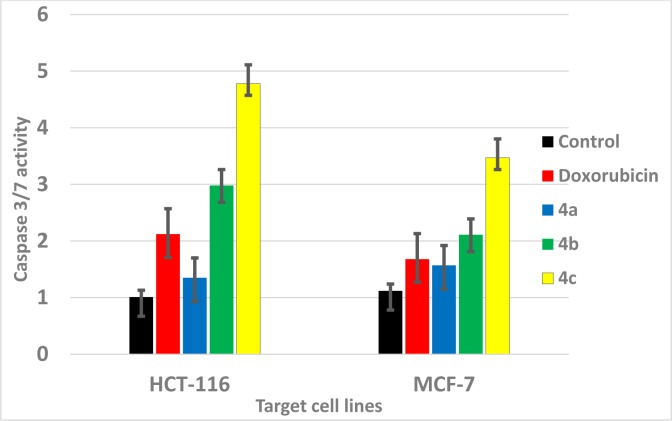
*In vitro* analysis of caspases activity for representative target compounds

The initial reported assay of 4-aryl-4*H*-chromene series were identified as apoptosis inducers via caspase activation (Elinson et al., 2010[20]; Sondhi et al., 2010[58]; Thareja et al., 2010[60]; Trinchieri, 2015[61]). Furthermore, it was important to investigate the antiproliferative effect of the novel synthesized compounds **4a, 4b, **and **4c **using caspase-3 and -7 activities compared to doxorubicin and vehicle control drug in the corresponding cancer cell lines MCF-7 and HCT-116. The resulting data are presented in Figure 8(Fig. 8). The results indicated that all tested compounds significantly increased the concentration of caspases compared to doxorubicin and vehicle control. In particular, compound **4b** and **4c **showed significant elevation in the caspase activity compared to control and doxorubicin drug by 2-3 folds. These results suggested that the antiproliferative effect of these derivatives is mediated through induction of apoptosis.

#### In vitro inhibitory activity screening of E. coli DNA gyrase

Based upon the antimicrobial screening data, target compounds **4a**, **7c**, and **13b **were evaluated against DNA gyrase from *Escherichia coli *in the DNA supercoiling assay for studying their mechanism. The results are presented in Table 5(Tab. 5) as residual activities (RA is the percentage activity of the enzyme in the presence of 10 μM and 100 μM of compound). Unfortunately, all tested compounds displayed weak activity as DNA gyrase inhibitor by 114 % to 137 % residual activities. This means that the chromene and azo chromene compounds did not affect the antimicrobial activity through this enzyme target.

#### Acute toxicity

**Figure 9:**
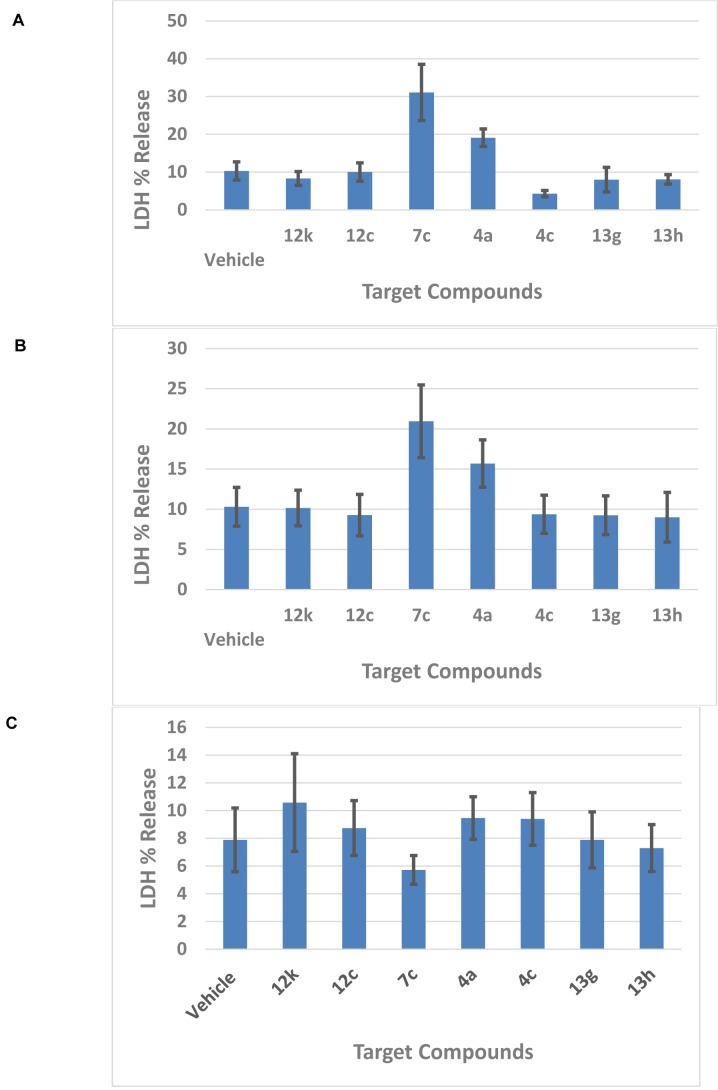
Bar chart summarizing the effect of compounds on acute neuronal toxicity. These graphs show the acute toxicity of target compounds against neuronal cells. A) The reported toxicity of target drugs within 24 h by concentration of 10 µM, B) The reported toxicity of target drugs within 24 h by concentration of 100 µM, C) The reported toxicity of compounds within 48 h (10 µM). The data represent mean with standard error values.

Lactate dehydrogenase (LDH) release in the medium is an enzymatic indicator that illustrates the loss of membrane integrity, apoptosis, or necrosis of a cell. Using a primary mixed neuronal *in vitro *culture, the cytotoxic effect of the chromene-based compounds (**4a, 4c, 7c, 12c, 12k, 13g**, and **13h**) was assessed by lactate dehydrogenase (LDH) release from cells pretreated with different concentrations after 24 and 48 h of incubation (Figure 9A-C(Fig. 9)). It was shown that after 24 hrs of incubation and using 10 µM concentration of drugs, compound **7c** and **4a** exhibited more LDH release than vehicle by 2-3 folds, while compound **4c** reduced the LDH release (Figure 9A(Fig. 9)). By increasing the concentration to 100 µM for 24 hrs, only compound **7c** displayed more LDH release compared to the vehicle, while compound **4c** was ineffective at this concentration (Figure 9B(Fig. 9)). Increasing the incubation time of the compounds to 48 hrs completely abolished the effect of these compounds (Figure 9C(Fig. 9)). These results confirmed that compounds **7c** and **4a** have a cytotoxic effect of on central nervous system tissue.

### Qualitative complex metric analysis

**Figure 10:**
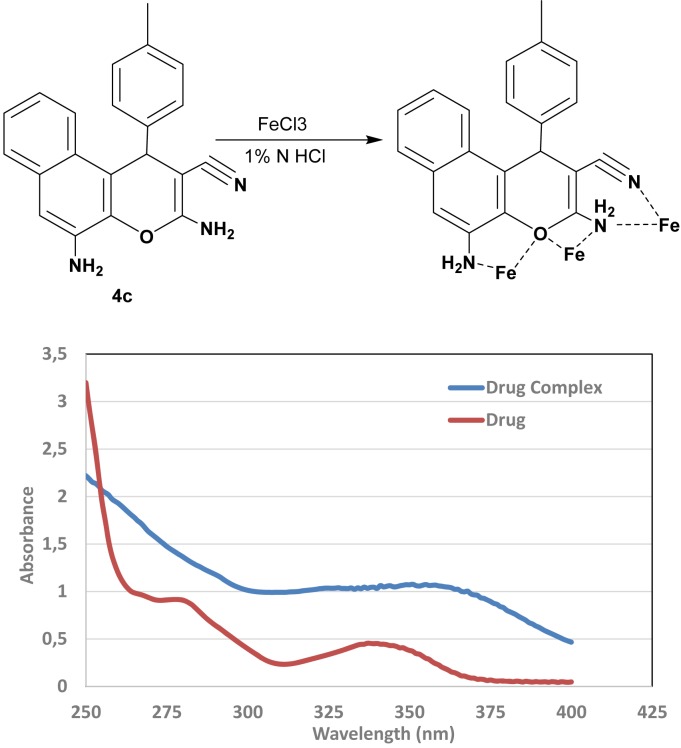
UV-vis study for metal chelation with target compound 4c

The tendency of the target drugs to work through a chelation mechanism with metals like iron and calcium, which would be similar to the behavior of doxorubicin as reference drug, was performed experimentally. The experiment was done by reaction of ferric chloride as metal source and compound **4c** as a representative of one of our target compounds. The data are presented in Figure 10(Fig. 10), showing an absorption peak at λ_max _equal 361 nm which is shifted bathochromically due to the complexation. The absorption spectrum was observed to change from 281 and 342 nm for the drug alone, to 361 nm for the drug complex. This behavior occurs due to the possibility of monocomplexation, dicomplexation or tricomplexation reactions and indicates that these chemical compounds have the ability to chelate with metals as part of their biological cytotoxic mechanism. 

### Computational studies and SAR analysis

#### 2D-QSAR analysis

**Figure 11:**
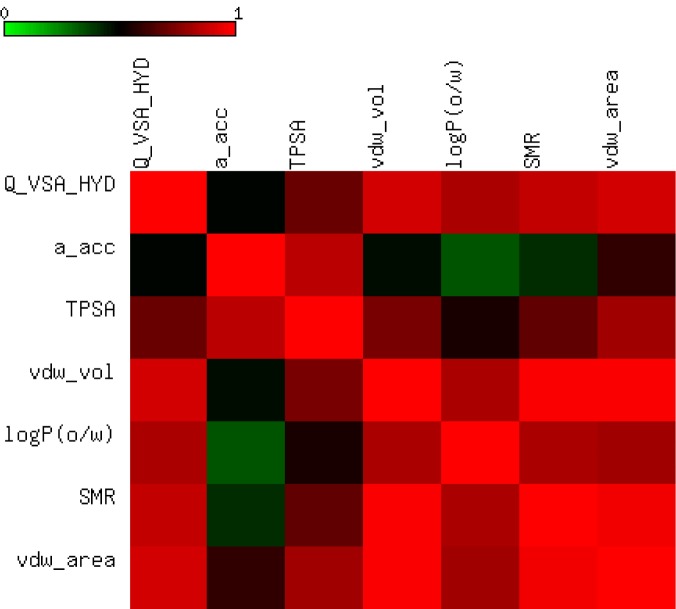
Heat map of correlation matrix of descriptor values

**Figure 12:**
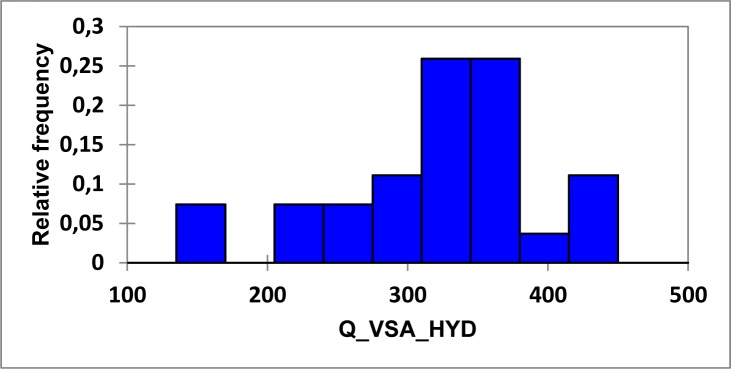
Histogram distribution of target compounds according to Q_VSA_HYD values

**Figure 13:**
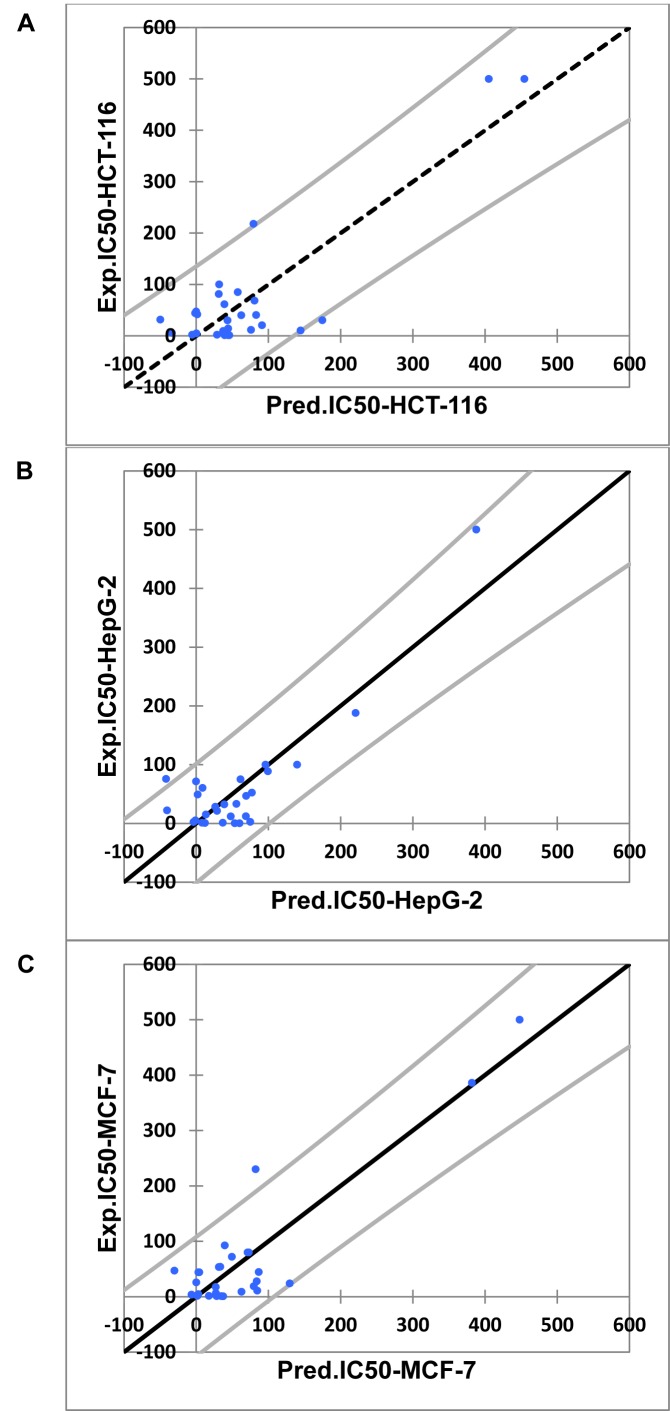
QSAR plot of correlation of the observed against predicted IC_50_ of A) HCT-116, B) HepG-2, C) MCF-7 cell lines

In this study, the best multi-linear regression analysis was utilized a stepwise search tool for finding out n-parameter regression models (n is the number of descriptors) for relating of structures to activity based upon different parameters coefficients values like (correlation coefficients, R^2^), F value, and standard deviation. Three 2D QSAR equations were built using a set of 28 synthesized compounds with regard to each showing anticancer bioactivity in terms of molecular descriptors. These descriptor panels include: total polar surface area (TPSA), van der Waals volume (vdw_vol), partition coefficient (logP(o/w), number of hydrogen bonding acceptor (a_acc), total hydrophobicity surface (Q_VSA_HYD), molar refractivity (SMR), and van der Waals surface area (vdw_area). The QSAR models were generated from up to 6 descriptors describing the biological activity of anticancer agents and agree with the rule of thumb; 5:1 ratio of compounds to descriptors. The statistical characteristics of all models are grouped in Table 6(Tab. 6). It is quite clear that the models are significant based on statistical parameters. Table 7(Tab. 7) reports the distribution values of descriptors space, while Table 8(Tab. 8) and Figure 11(Fig. 11) account for the least correlation analysis among them. The frequency of compound set distribution based upon Q_VSA_HYD descriptor is sketched in Figure 12(Fig. 12), showing the differential properties among novel derivatives. Figure 13(Fig. 13) shows the QSAR plots of correlations representing the observed versus predicted IC_50_ for all antiproliferative screening. The observed and predicted data of the training set of compounds (**1, 4a-I, 5, 7a-e, 12c-k, **and** 13b-k**), with exception of **4e, 4i, 7c, 13b**, and **13f **as testing sets, are presented in Table 7(Tab. 7). 

#### Docking simulations

**Figure 14:**
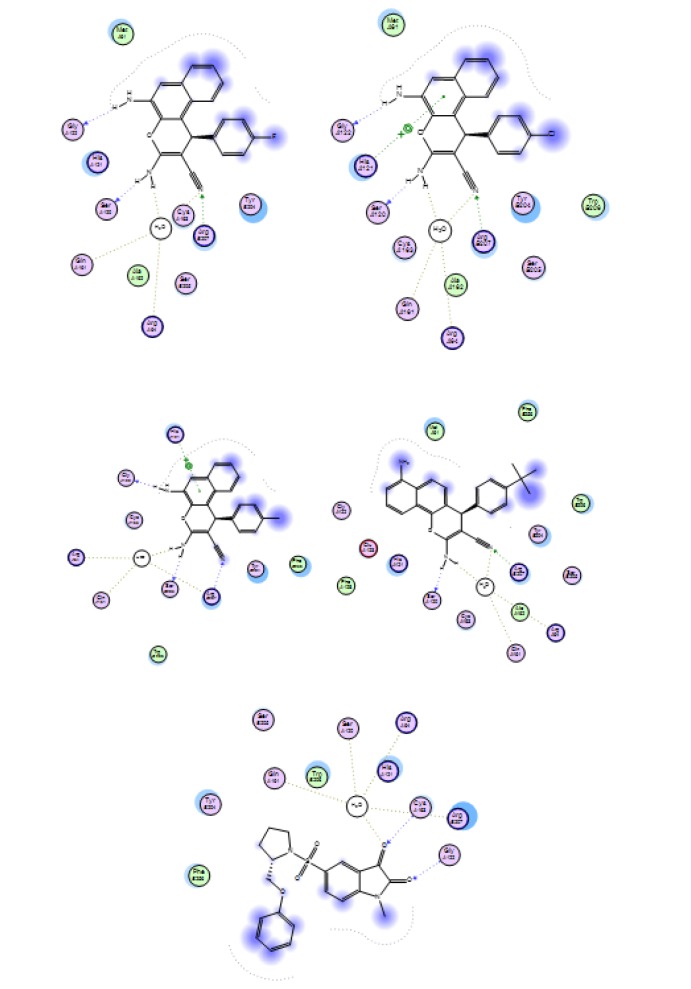
Molecular interactions of the target compounds 4a, 4b, 4c, 7c, and reference ligand with active site of target protein caspase-3 enzyme

**Figure 15:**
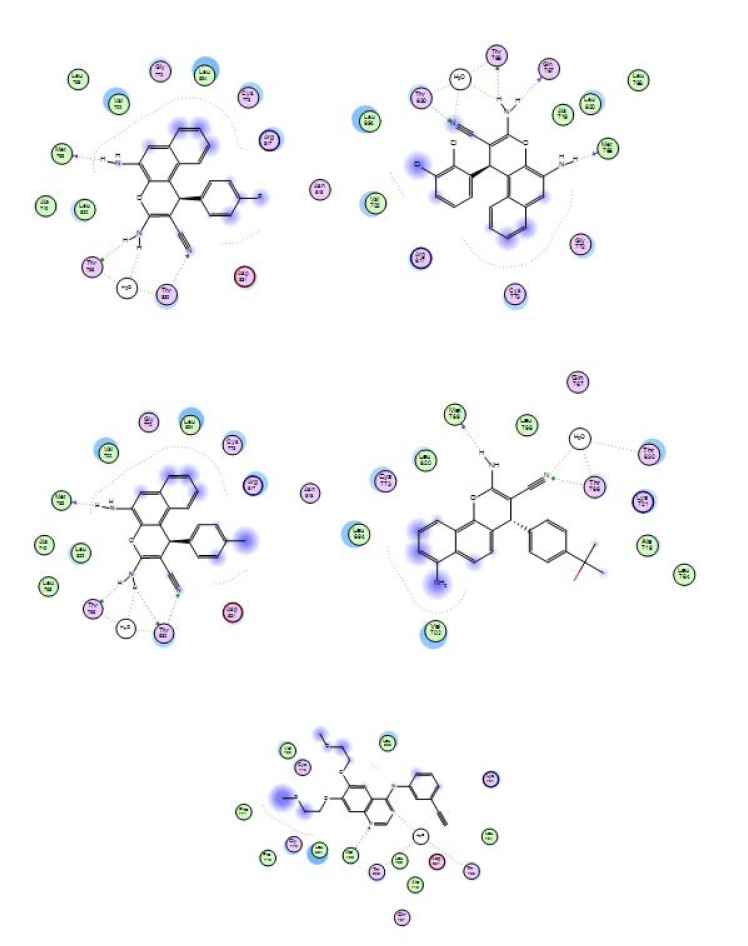
Molecular interactions of the target compounds 4a, 4b, 4c, 7c, and reference ligand with active site of target protein EGFR enzyme

Molecular docking studies are one of the main challenging aspects in the computer drug discovery processes. Docking analysis was applied for the potent compounds of the two series **4a-c** and **7c** for discovery of their mode of action. The process was done with the selective pharmacological target protein which regulates the cell proliferation at various stages in breast and colon cancers. The inverse docking process has demonstrated that the proteins, Caspase-3 and EGFR have significant docking score and binding affinity with corresponding compounds. The H-bond profile and binding affinity of compounds towards active site amino acids of target proteins were presented in Figures 14(Fig. 14) and 15(Fig. 15). According to the results of compounds docking, they behaved as the original reference drugs do in the protein pocket. In case of caspase-3 enzyme, all three potent compounds **4a-c **formed stable hydrogen bonding system with the targets through the triad fragments in their structures, NH_2_-NH_2_-CN by the corresponding amino acid residues **Gly122**, **Ser120**, **Arg207**, and **Gln161**. In addition, a hydrophobic contact with the rest of the pocket had occurred by the substituted phenyl ring. For EGFR target, the triad fragment system NH_2_-NH_2_-CN also formed stable hydrogen bonds with the amino acid **Met769**, **Gln767**, **Thr766**, and **Thr830** residues in the pocket. As well, arene aromatic interaction occurred with target residues through the substituted phenyl ring. In contrast to compound **7c**, the amino group is so far from the rest of triad fragment system and hence lost the hydrogen bonding interaction; this is what made the activity different. This interaction analysis is consistent with the reference drug interaction and what was published (Ruddarraju et al., 2016[49]). The behavior of these ligands against target proteins explained the importance of the presence of amino group to be neighbor to the hydroxyl group and the size substitution on phenyl ring. The anti-cancer activity of the novel synthesized compounds and their molecular interactions with various therapeutic targets related to cancer in the docking study proved that more number of pharmacophore features (H-bonds acceptors, H-bond acceptor and Donors, Aromatic centers) in the substructures of chromene nucleus played a crucial role in the formation of H-bonding and aromatic interactions with functional groups of inhibitor binding site residues leading to down regulation of cancer cell signaling or apoptotic activation.

## Conclusions

New series of chromene compounds have been prepared via multi-components reaction of 3-amino-2-naphthol with malononitrile and aromatic aldehydes in the presence of piperidine. The structures of the obtained compounds were confirmed using FT-IR, NMR and mass spectroscopy. Using the same methodology, a novel series of chromene containing azo chromophores was successfully synthesized via two steps' reaction. First, preparing the azo dye derivatives followed by the established methodology of the multi-components chromene approach. UV-vis of the new materials shows that the incorporation of the chromene moieties didn't alter their λ_max_ values. The new compounds have been evaluated for their antimicrobial and antiproliferative activities. Several of the target compounds showed potent antibacterial and antifungal effects towards some strains. In addition, they displayed more potency as anticancer agents with IC_50_ values in low micromolar concentrations. Certain studies have been employed to clarify their mechanisms and proved that these series could play their rule through the apoptotic effect by activation of caspases. As well, SAR analyses were discussed and explained by application of different computational methods.

## Acknowledgement

The authors would like to express their appreciation to the Deanship of Scientific Research at Taibah University, Al-Madinah Al-Munawarah, Saudi Arabia for financial project support 6305/1435.

## Declaration of interest

The authors declare no conflicts of interest.

## Figures and Tables

**Table 1:**
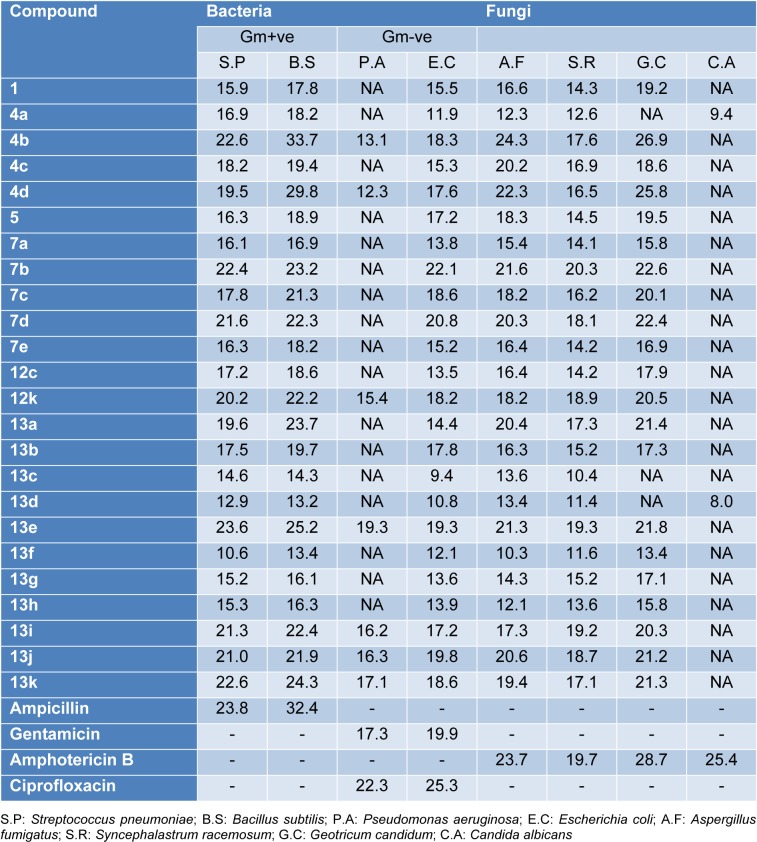
Well diffusion assay for antimicrobial activity of synthetic compounds (Inhibition Zone (IZ) diameter in mm) (5 mg/mL in DMSO)

**Table 2:**
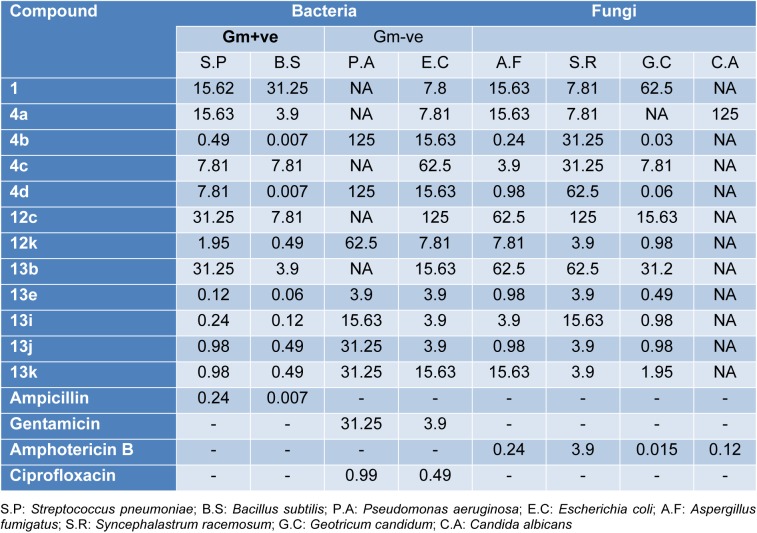
Antimicrobial activity of synthetic compounds (Minimum inhibitory concentration, MIC, µg/mL)

**Table 3:**
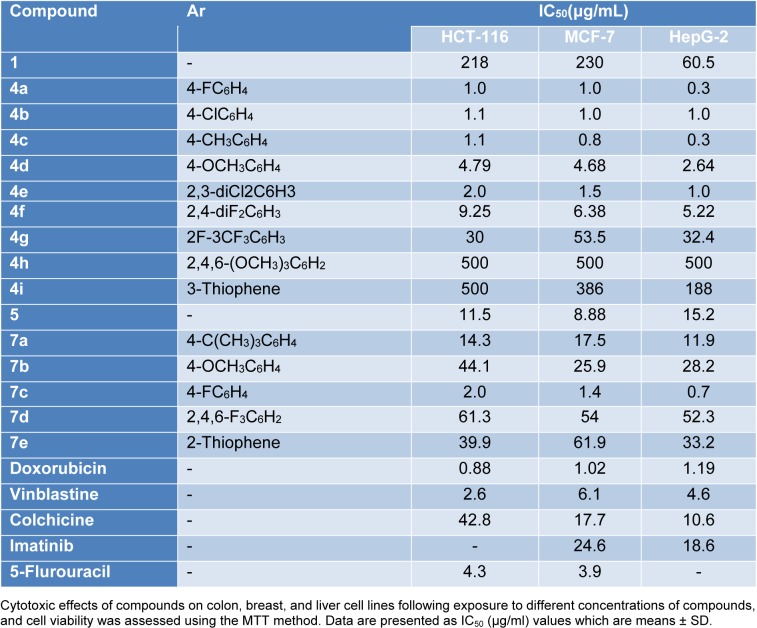
Cytotoxicity of target Benzo Chromene compounds against three different cancer cell lines

**Table 4:**
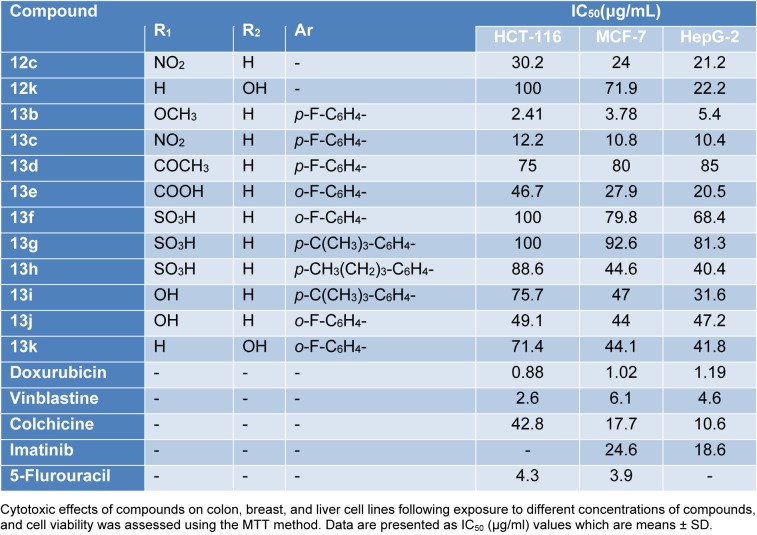
Cytotoxicity of target azo dyes and azo based chromene compounds against three different cancer cell lines

**Table 5:**
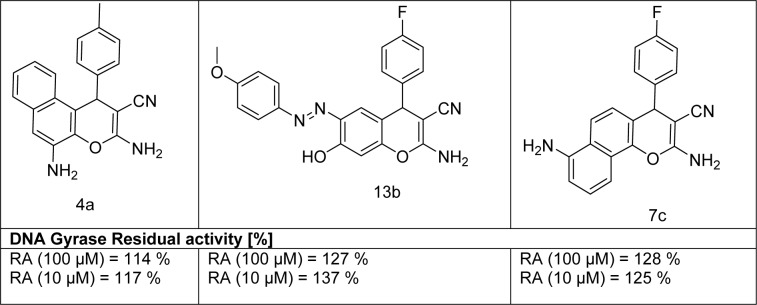
DNA Gyrase data for some selected chromene and azo based chromene compounds

**Table 6:**
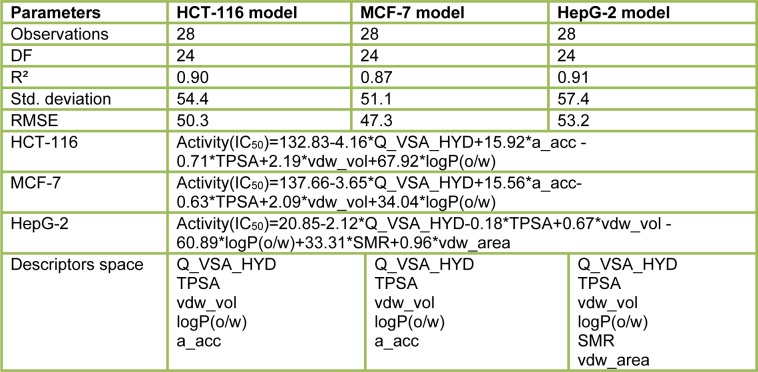
QSAR models with all details including equations, descriptors space, and correlation parameters

**Table 7:**
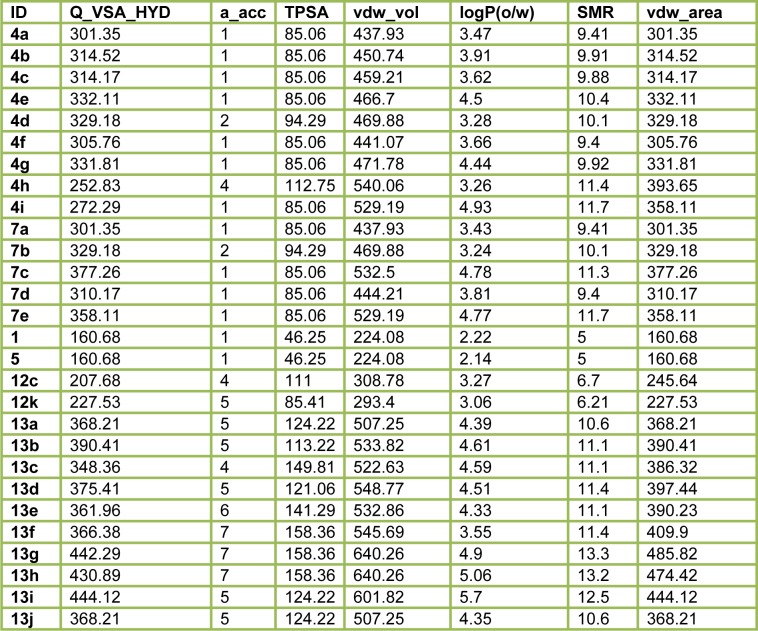
Molecular descriptor values of the QSAR models

**Table 8:**
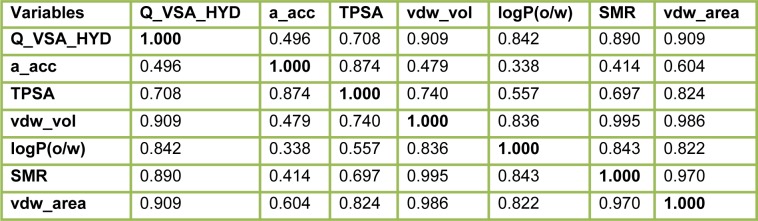
Correlation matrix of descriptor values
